# Nanomedicine as a Promising Tool to Overcome Immune Escape in Breast Cancer

**DOI:** 10.3390/pharmaceutics14030505

**Published:** 2022-02-25

**Authors:** Alba Navarro-Ocón, Jose L. Blaya-Cánovas, Araceli López-Tejada, Isabel Blancas, Rosario M. Sánchez-Martín, María J. Garrido, Carmen Griñán-Lisón, Jesús Calahorra, Francisca E. Cara, Francisco Ruiz-Cabello, Juan A. Marchal, Natalia Aptsiauri, Sergio Granados-Principal

**Affiliations:** 1 GENYO, Centre for Genomics and Oncological Research, Pfizer/University of Granada/Andalusian Regional Government, 18016 Granada, Spain; albanaoc@correo.ugr.es (A.N.-O.); jose.blaya@genyo.es (J.L.B.-C.); araceli.lopez@genyo.es (A.L.-T.); rosario.sanchez@genyo.es (R.M.S.-M.); carmen.grinan@genyo.es (C.G.-L.); jesus.calahorra@genyo.es (J.C.); francisca.cara@genyo.es (F.E.C.); 2 Instituto de Investigación Biosanitaria (ibs.GRANADA), 18012 Granada, Spain; iblancas@ugr.es (I.B.); fruizc@ugr.es (F.R.-C.); jmarchal@ugr.es (J.A.M.); 3 UGC de Oncología Médica, Complejo Hospitalario de Jaen, 23007 Jaen, Spain; 4 Department of Biochemistry and Molecular Biology 2, School of Pharmacy, University of Granada, 18011 Granada, Spain; 5 UGC de Oncología, Hospital Universitario “San Cecilio”, 18016 Granada, Spain; 6 Department of Pharmaceutical Technology and Chemistry, School of Pharmacy & Nutrition, Navarra Institute for Health Research (IdisNA), University of Navarra, 31080 Pamplona, Spain; mgarrido@unav.es; 7 Department of Biochemistry, Molecular Biology 3 and Immunology, School of Medicine, University of Granada, 18071 Granada, Spain; 8 Department of Human Anatomy and Embryology, School of Medicine, University of Granada, 18016 Granada, Spain

**Keywords:** breast cancer, nanomedicine, immune escape, cancer immunotherapy, cancer treatment

## Abstract

Breast cancer is the most common type of malignancy and leading cause of cancer death among women worldwide. Despite the current revolutionary advances in the field of cancer immunotherapy, clinical response in breast cancer is frequently below expectations, in part due to various mechanisms of cancer immune escape that produce tumor variants that are resistant to treatment. Thus, a further understanding of the molecular events underlying immune evasion in breast cancer may guarantee a significant improvement in the clinical success of immunotherapy. Furthermore, nanomedicine provides a promising opportunity to enhance the efficacy of cancer immunotherapy by improving the delivery, retention and release of immunostimulatory agents in targeted cells and tumor tissues. Hence, it can be used to overcome tumor immune escape and increase tumor rejection in numerous malignancies, including breast cancer. In this review, we summarize the current status and emerging trends in nanomedicine-based strategies targeting cancer immune evasion and modulating the immunosuppressive tumor microenvironment, including the inhibition of immunosuppressive cells in the tumor area, the activation of dendritic cells and the stimulation of the specific antitumor T-cell response.

## 1. Introduction

Breast cancer is the most common malignancy among women worldwide, showing an incidence rate of 10.4% of all cancers. It is the leading cause of death for women aged between 20 and 59 years and it is responsible for 15% of deaths from cancer, being the second cause around the world. On the other hand, overall survival of patients with breast cancer has significantly improved in recent years due to the earlier detection of the disease and the optimization of treatment regimens [1,2]. Immunotherapy is one of the most promising novel strategies to treat different forms of malignancies, including breast cancer [3,4,5]. Indeed, it has provided remarkable clinical outcomes in cancer patients and has marked a milestone in the treatment of the disease [6]. This approach to eliminating cancer cells is based on boosting the intrinsic mechanisms of the immune system to specifically recognize and destroy malignant cells and improve the antitumor immune response in patients [7].

Targeting the immune system has currently become a key approach to treat many types of cancer. In fact, several strategies based on immunotherapy have recently been developed to treat breast cancer, with encouraging clinical outcomes [8,9,10]. However, the efficacy of cancer immunotherapy for breast cancer is not yet as satisfying as it was expected to be because treatment response rates in patients vary among different subtypes of breast cancer and within cohorts with the same malignancies [11,12]. The main drawbacks that limit the clinical success of immunotherapy are the mechanisms that cancer cells develop to escape from immune surveillance and the immunosuppressive tumor microenvironment (TME) that they create as a result. Moreover, breast cancer particularly involves some important challenges for immunotherapy treatments. Breast tumors have especially low immunogenicity, with the exception of triple-negative breast cancer (TNBC) and HER2+ subtypes. In comparison with other tumors, the burden of nonsynonymous DNA mutations in breast cancer is relatively poor; thus, the tumor major histocompatibility complex (MHC) exhibits lower numbers of neoepitopes (mutant cancer antigens) to the effector immune cells. In this way, the immunogenicity of breast tumors is poor and the antitumor T-cell reactivity is modest [13]. In addition, most subtypes of breast cancer show low tumor infiltration with lymphocytes because of the immunosuppressive TME, which in turn is linked to a worse prognosis [14,15,16,17]. Therefore, boosting the antitumor immune response in breast cancer is remarkably challenging. In this regard, improving cancer immunotherapy remains a high priority nowadays, since it may provide a remarkable increase in overall survival of those afflicted with breast cancer and an effective long-term antitumor immunity [18]. One of the best strategies to achieve better clinical outcomes of cancer immunotherapy is to overcome the mechanisms of immune escape that are developed by cancer cells. Moreover, using nanomedicine to improve the administration and/or efficacy of immunotherapy is an excellent approach to make progress in the treatment of this disease. Nanoparticles are able to overcome physical and biological obstacles in the delivery of the immunomodulating therapy, especially in solid tumors, as they can modify the TME to increase tumor immune infiltration, as well as targeting and penetrating solid tumors [19]. Furthermore, the systemic toxicity and the risk of adverse effects are also minimized when nanomedicine is employed. Hence, in the present review, we first describe the key mechanisms of breast cancer immune escape and, secondly, we focus on the current most relevant nanomedicine-based approaches in cancer immunotherapy to overcome immune evasion and improve conventional treatment in breast cancer.

## 2. Mechanisms of Cancer Immune Escape

Immune system exerts host-protecting actions on developing tumors by eliminating malignant cells. However, cancer cells often develop mechanisms of immune escape that limit the ability of innate and adaptive immunity responses. These mechanisms truly predetermine both cancer progression and the response of patients to cancer immunotherapy protocols [20,21,22]. Therefore, a better understanding of these mechanisms may open the door to innovative and more efficient therapies for cancer. Here, we describe some of the most relevant mechanisms of immune escape in breast cancer.

### 2.1. Tumor-Intrinsic Mechanisms of Immune Evasion

Cancer cells usually exhibit alterations in human leukocyte antigen class I (HLA-I) expression, overexpress programmed cell death ligand 1 (PD-L1) and release immunosuppressive cytokines into the TME.

#### 2.1.1. Alterations in Tumor HLA-I Expression

The HLA-I complex often is downregulated or absent in malignant cells to avoid the immune response. Indeed, altered HLA-I phenotypes have been highly frequently detected in patients with invasive breast carcinomas [23]. HLA-I alterations can be classified in two main groups: irreversible structural or reversible regulatory defects. On the one hand, the most important mechanisms that lead to irreversible defects of HLA-I are loss of heterozygosity (LOH) at chromosome 6 (harboring *HLA-A,B,C* genes) and LOH at chromosome 15 (carrying the *beta-2 microglobulin* gene) [24]; both of them have a high incidence in breast cancer [25]. On the other hand, reversible downregulation of HLA-I expression can be caused by the epigenetic downregulation of HLA-I heavy-chain genes, the *beta-2 microglobulin* gene or any component of the antigen processing and presentation machinery (APM) [26]. In any case, the reversible defects can be corrected by different therapies, such as the administration of interferon gamma (IFN-γ) or other cytokines that are commonly induced by immunotherapy [24].

#### 2.1.2. Overexpression of PD-L1

Cancer cells commonly overexpress the inhibitory PD-L1 on their surface. Hence, tumor cells are able to inactivate T cells in the tumor area and suppress the antitumor immune activity [27,28]. Indeed, high levels of PD-L1 expression in cancer cells have been found in various types of breast cancer, including small-cell breast carcinomas, basal tumors and inflammatory breast cancers [29,30].

#### 2.1.3. Production of Immunosuppressive Cytokines

Cancer cells produce cytokines and chemokines that can influence the maturation and antitumor function of immune cells. In patients with breast cancer, increased plasma concentrations of vascular endothelial growth factor (VEGF) were associated with a decrease in the function and density of mature dendritic cells (DCs). This finding has been associated with increased serum concentrations of IL-10, which also compromises the functionality of DCs because it hampers their maturation and enhances their spontaneous apoptosis. In addition, IL-10 inhibits the induction of the tumor-specific T helper type 1 (Th1) cells, the production of IL-12 by macrophages and the antigen presentation [31]. Furthermore, transforming growth factor-beta (TGF-β) is frequently overproduced by tumor cells and found in high concentrations in breast cancer patients. TGF-β enhances macrophages’ production of IL-10 and drives the shift in the Th1/T helper type 2 (Th2) balance towards the anti-inflammatory and tumor-promoting Th2 response. Therefore, TGF-β is associated with disease progression and poor responses to immunotherapy in breast cancer patients [32]. Furthermore, breast tumors secrete the soluble MHC-I polypeptide-related sequence A (MICA) protein that binds to the activating natural killer group 2 member D (NKG2D) receptor and attenuates the cytotoxicity of natural killer (NK) cells [33]. Tumor cells are also able to escape from NK lysis due to their lack of expression of costimulatory molecules, such as B7-1 (also known as CD80), B7-2 (or CD86), CD40 and CD70, that hinder the optimal activation of these cells. Additionally, TGF-β and other immunomodulatory cytokines produced by tumor cells are able to inhibit NK cells’ activation and function [34].

Finally, most human tumors also overproduce galectin-1 (Gal1) and indoleamine 2,3-dioxygenase (IDO). On the one hand, Gal1 blocks the antitumor function of CD8+ T cells and promotes the differentiation of highly suppressive regulatory T cells (Tregs) [35]. It was identified as an abundant protein in breast tumors and correlates with aggressiveness and metastasis of breast cancer because it enhances the pro-tumor Th2 response and triggers the expansion of CD4+ CD25+ Foxp3+ Tregs cells [36]. On the other hand, IDO provokes the proliferation arrest of alloreactive T lymphocytes [37]. In fact, high levels of expression of IDO and Tregs were observed within sentinel lymph node metastases in many breast cancer patients [38].

### 2.2. Immunosuppressive Cells in Tumor Microenvironment

Increased numbers of both CD4+ CD25+ Tregs and myeloid-derived suppressor cells (MDSCs) were found in the peripheral blood of breast cancer patients [39]. Tregs play a key role in immunosuppression because they can block the antitumor immune response by inhibiting the proliferation/activation of cytotoxic T lymphocytes (CTLs) and the secretion of Th1 cytokines, as well as stimulating the recruitment of MDSCs [40]. Tumor-infiltrating MDSCs can inhibit mature DCs and modulate the activation of T cells due to their immunosuppressive ability. Among stromal elements, carcinoma-associated fibroblasts (CAFs) also play a significant role in breast cancer. They are also involved in immune escape due to their secretion of various soluble immunomodulatory factors, such as IL-1 and TGF-β. In breast carcinomas, around 80% of stromal fibroblasts have the CAF phenotype [41,42]. In addition, breast cancer patients have reduced numbers and functioning of peripheral blood and sentinel lymph node DCs [31]. These DCs exhibit a diminished secretion of inflammatory IL-12 and low surface expression of HLA-II and CD80/CD86 molecules. Moreover, the metastatic dissemination of breast cancer to tumor-draining lymph nodes is associated with the apoptosis and maturation arrest of DCs and a lack of direct contact with CD8+ T lymphocytes. Hence, CTLs cannot be activated by DCs to eliminate tumor cells [34,43].

Lastly, IL-4-expressing CD4+ T cells were found to directly regulate tumor-associated macrophages (TAMs) in preclinical breast cancer models [44]. Macrophages are classified into the classically (M1) and the alternatively (M2) activated phenotypes. M1 macrophages contribute to an efficient antigen presentation, promote the Th1 cell response and release pro-inflammatory cytokines, whereas M2 macrophages produce low amounts of pro-inflammatory cytokines and high amounts of the anti-inflammatory cytokine IL-10 [45]. TAMs exhibit the M2 phenotype in most malignancies, which contributes to tumor growth and dissemination. Hence, the density of TAMs was found to be correlated with a poor prognosis in human breast carcinomas [46].

### 2.3. Impairment of Cytotoxic T-Cell Immunity

Many of the previously mentioned mechanisms of immune evasion indirectly alter the anti-tumor cytotoxicity of T cells. TGF-β and IL-10 production in the TME downregulate the antitumor Th1 cellular responses and inhibit the lytic function of CTLs. Furthermore, alterations in the antigen presentation and activation of antigen-presenting cells (APCs), as well as overexpression PD-L1 by tumor cells, prevent T lymphocytes’ activation.

Importantly, tumor cells are also able to directly inhibit T-cell activity by different mechanisms. They often exhibit surface expression of chemokine ligand 9 (CXCL9) and 10 (CXCL10), which impair the recruitment of T cells into the tumor area [47]. Tumor cells frequently have an increased B7-H1 (PD-L1) and B7-H4 expression, causing inhibition of T lymphocyte activation. Indeed, overexpression of B7-H4 has been reported in most breast carcinomas [48]. Moreover, tumor cells often show a downregulation of costimulatory CD80/CD86 molecules, which hampers T cells’ activation and contributes to low T-cell function [47]. On the other hand, tumor-infiltrating CD8+ T cells frequently have an exhaustion phenotype with altered clonal expansion and reduced cytotoxicity and production of inflammatory cytokines [49]. In addition, the overexpression of Fas (CD95) by T lymphocytes and the Fas ligand (FasL) by tumor cells induces the apoptosis of activated T cells [50]. Furthermore, in breast cancer patients, cytotoxic CD8+ T cells display a significant downregulation of the T-cell receptor (TCR) ζ chain and CD28, which causes T-cell anergy and death [51]. All these changes together reduce the number of activated tumor-specific effector cells in breast cancer and produce immunosuppressive TME.

## 3. Nanomedicine as a Tool to Overcome Mechanisms of Cancer Immune Escape: A Promising Strategy to Treat Breast Cancer

During the last decade, nanotherapy has been gaining relevance in the immunotherapy field because of its numerous advantages over conventional therapies. The size and adjustable surface properties of nanoparticles (NPs) provide useful features for treatments. For instance, the high biocompatibility and stability of nanocarriers enhance the blood circulation of drugs. Furthermore, nanosystems exhibit a greater accumulation in tumor tissues than traditional treatments since they are sufficiently small to overcome physical and biological barriers and extravasate by passive targeting, which is known as the enhanced permeation and retention (EPR) effect [52,53,54]. In addition, NPs possess remarkable tumor specificity as they are able to accommodate multiple ligands to be delivered into the tumor area by active targeting. Hence, cancer nanomedicine exhibits higher therapeutic efficacy and reduces the systemic toxicity of carried drugs. On the other hand, nanoformulations are able to carry large loads and allow the administration of different cancer drugs in the same application to enhance their antitumor effects. Moreover, NP-based strategies in several mouse breast cancer models were able to convert poorly immunogenic or “cold” tumors into immunogenic or “hot” tumors, establish a T-cell memory immune response, inhibit tumor growth and metastasis and prevent tumor relapse [19]. Thus, many novel nanomedicine-based therapies have been proposed to alleviate immunosuppression in tumors and reduce the emergence of tumor escape variants in patients with breast cancer (Table 1).

### 3.1. Applications of Nanomedicine to Target Immunosuppressive Tumor Metabolism and Immunosupressive Cytokines in the TME

IL-10 is an immunosuppressive cytokine with an important role in the TME and high levels of serous IL-10 have been associated with poor survival in breast cancer patients. Thus, targeting IL-10 has the potential of becoming an effective therapy for this disease. In this context, lipid-protamine-DNA (LPD) NP platform was loaded with plasmids encoding small antibody-like IL-10 protein trap and locally delivered into a murine 4T1 TNBC model. This IL-10 protein trap caused a regional and transient reduction in IL-10 production and an increased expression of proinflammatory cytokines TNF-α and IFN-γ in the TME. Hence, the IL-10 trap significantly inhibited tumor growth by decreasing M2 macrophages, MDSCs and TFG-β production in the tumor, and enhanced the median survival rate in the in vivo model [55].

**Figure 1:**
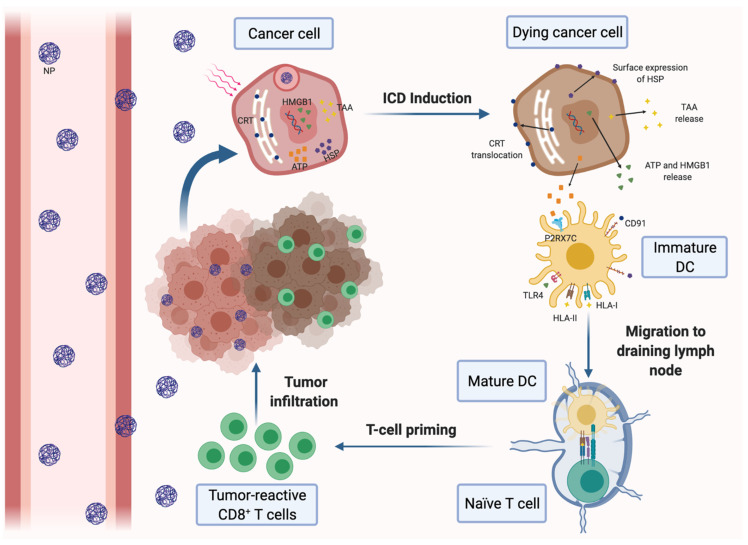
Induction of immunogenic cell death (ICD) of cancer cells by nanomedicine-based photodynamic or photothermal therapy. Nanoparticles (NPs) carrying a photodynamic agent extravasate blood vessels, reach breast tumors and are internalized by cancer cells, where they release their loading. Upon laser irradiation, the dying cancer cells secrete tumor-associated antigens (TAAs) and damage-associated molecular patterns (DAMPs), such as ATP and high-mobility group box 1 protein (HMGB-1). Tumor cells also express DAMPs on the cell surface, including calreticulin (CRT) and heat-shock proteins (HSP). DAMPs can be recognized by different pattern recognition receptors expressed on dendritic cells (DCs), resulting in their maturation and activation. Mature DCs migrate to the draining lymph nodes and induce the activation of tumor-specific effector CD8+ and CD4+ T cells, which infiltrate the tumor and mediate the specific antitumor immune response.

Growing tumors are also characterized by vascular abnormalities, high lactate levels and glucose deprivation, which lead to acidic pH and hypoxia conditions within the tumor. Both factors have immunosuppressive effects and significantly affect the clinical outcomes in many cancer therapies [134,135]. For that reason, nanomedicine has also been used to regulate hypoxia and acidity in the TME. Novel designed biodegradable hollow manganese dioxide (H-MnO2) nanoshells were co-loaded with the photodynamic agent chlorine e6 (Ce6) and the chemotherapy drug doxorubicin (Dox), generating a nanoplatform (H-MnO2-PEG/C&D) that is dissociated under reduced pH levels within the TME to specifically release loaded drugs or cargo in the tumor area. Furthermore, this nanoplatform could reduce local tumor hypoxia by inducing decomposition of tumor endogenous H_2_O_2_ into water and oxygen and further promote photodynamic therapy (PDT), in which oxygen is actively involved. In this way, after systemic injection of H-MnO2-PEG/C&D into 4T1 tumor-bearing mice and light exposure, tumor-associated Tregs population was reduced, while tumor infiltration of CTLs and TAMs was significantly increased with a notably M1 polarization. More significantly, a combined treatment with this nanoplatform and PD-L1 blockade potentiated antigen-specific CTLs and inhibited the growth of light-irradiated primary tumors cells as well as protecting against light-exposure tumors [56]. Similarly, core-shell gold nanocage@manganese dioxide (AuNC@MnO2, AM) NPs have been developed to increase oxygen concentration in the TME, with the aim of enhancing the PDT treatment in a metastatic TNBC murine model. The acidic pH of the TME breaks down the nanoplatform shell and NPs generate a massive release of oxygen in the tumor area. In turn, oxygen-boosted PDT elicits the immunogenic cell death (ICD) of cancer cells (Figure 1), followed by the liberation of damage-associated molecular patterns (DAMPs) and their subsequent presentation by mature DCs to effector immune cells. Therefore, the nanoplatform combined with PDT in this model was able to induce a systemic antitumor immune response, destroy primary tumors and prevent tumor metastases [57].

In another approach, small interference RNA (siRNA) cationic lipid-assisted NPs (CLAN) were designed to neutralize tumor acidity by inactivating lactate dehydrogenase A (LDHA). In cancer cells, LDHA converts pyruvate to lactic acid because of elevated tumor aerobic glycolysis, which leads to the pH decreasing in the TME. Tumor acidity promotes CD8+ T cells apoptosis and anergy, while high levels of lactate enhance the function of MDSCs and Tregs and polarize TAMs to the M2 phenotype. siRNA CLAN were systematically injected into a murine 4T1 mammary tumor model to downregulate lactate production and neutralize tumor pH before the administration of an anti-PD-1 antibody. Hence, the efficacy of PD-1 blockade therapy was potentiated because tumor infiltration with CD8+ T and NK cells was increased and their functions were restored, whereas tumor-associated Treg density was reduced [58].

### 3.2. Applications of Nanomedicine to Target Immunosuppressive Cells within the TME

Nano-sized strategies have also been considered to inhibit the immunosuppressive effects of different immune cells that are abundantly found in the TME in breast cancer.

#### 3.2.1. Nanomedicine-Based Approaches for Targeting Neutrophils

Recently, cancer vaccination with virus-like particles (VLP), involving the spontaneous and non-infectious organization of viral coat proteins into the structure of a particular virus capsid, has been examined due to the intrinsic immunogenic properties that stimulate the immune response. In this way, an empty cowpea mosaic virus (eCPMV) VLP system was administered by Lizzote and co-authors to a 4T1 BALB/c syngeneic breast cancer model by inhalation to act as an in-situ vaccine. This system demonstrated an antitumor effect based on the activation of tumor-infiltrating N1 neutrophils, which finally induced a systemic antitumor immunity. N1 neutrophils coordinate the adaptive immune response, produce pro-inflammatory cytokines, recruit T and NK cells and stimulate antitumor cytotoxicity. Upon treatment, both a significant delay of lung metastasis onset and an extended survival of animals were observed [136].

#### 3.2.2. Nanomedicine-Based Approaches for Targeting NK Cells

With the purpose of promote antitumor activity of NK cells in the immunosuppressive TME, treatment with extracellular vesicles (EVs) derived from NK cells was investigated by Zhu et al. In this case, EVs were isolated from human NK cells treated with IL-15 (NK-EVs_IL-15_) and incubated with different cancer cell lines, including the breast cancer cell line MDA-MB-231/F. The priming of NK cells with IL-15, a key cytokine that promotes NK cell proliferation and survival, enhances their cytotoxicity. It also increases the secretion of EVs and promotes the NK-EVs_IL-15_ immunotherapeutic effects due to increased production of cytotoxic proteins (perforin and granzyme B), overexpression of the membrane Fas ligand (FasL) and improved tumor-targeting ability, when compared with EVs from untreated NK cells (NK-EVs). Moreover, NK-EVs_IL-15_ had a higher ability to induce apoptosis in cancer cells than NK-EVs and they were able to inhibit the growth of glioblastoma xenografts in mice [59].

#### 3.2.3. Nanomedicine-Based Approaches for Targeting Macrophages

TAMs have been reported to occupy up to 50% of breast tumors and most of them exhibit the M2 phenotype, and exert immunosuppressive and tumor-promoting effects. Thus, an important approach to battle cancer is to repolarize TAMs to the M1 phenotype, with immunostimulatory and tumor-inhibiting properties. Several strategies are currently being evaluated in order to achieve this goal in breast tumors. One of them consists in the systemic delivery of dual-pH responsive NPs that carry siRNAs targeting VEGF and placental growth factor (PIGF). Under acidic TME, both siRNAs were released and internalized into TAMs and breast cancer cells by mannose-mediated phagocytosis and passive targeting, respectively. These NPs downregulated VEGF and PIGF expression in cancer cells and M2-like TAMs, repolarized M2-TAMs to the M1 phenotype, reduced macrophage recruitment, angiogenesis and hypoxia within the TME and slowed down breast tumor growth and lung metastasis in the treated mice [137].

TAMs are polarized to the M2 phenotype upon the interaction between its colony-stimulating factor 1 receptor (CSF1R) and the macrophage colony-stimulating factor 1 (MCSF) produced by cancer cells. The binding of MCFS to CSF1R results in the activation of multiple downstream signaling pathways, including the mitogen-activated protein kinase (MAPK) pathway, which stimulates the proliferation and survival of M2-like TAMs. Therefore, another promising strategy to repolarize M2-like TAMs to the M1 phenotype could be based on the therapeutic vertical inhibition of the CSF1R and MAPK signaling pathways. Dual-kinase inhibitor-loaded supramolecular NPs that are able to co-deliver CSF1R and MAPK inhibitors into TAMs were used in an aggressive 4T1 breast cancer model and they enhanced the efficiency of M2-to-M1 repolarization, as well as anti-tumor efficacy [60]. Moreover, iron oxide NPs (ferumoxytol) were also demonstrated to induce M2-to-M1 repolarization of TAMs in the TME. These NPs were internalized by TAMs and increased the mRNA associated with Th1-type response, while the presence of M1 in the TME was augmented. Thus, antitumor cytotoxicity was improved and the growth of subcutaneous adenocarcinomas was inhibited in mice [61].

Another approach is based on the use of small molecule-gold nanorod conjugates to selectively target and induce macrophage cytotoxicity towards breast cancer cells in vitro. Gold NPs have been demonstrated to serve as targeted drug delivery vehicles and to act as contrast agents for near-infrared (NIR) laser photothermal tumor ablation. For that reason, colloidal gold nanorods (AuNRs) were functionalized with macrolides, which are antibiotics that are targeted towards macrophages. Since solid tumors are highly infiltrated with macrophages, this approach may facilitate the preferential delivery of the nanoconjugates to the tumor site. In this experimental model, AuNRs were preferentially delivered into TAMs, induced their antitumor activity and cytotoxicity against breast adenocarcinoma cells in co-culture and increased the efficacy of laser photothermal therapy (PTT). In addition, cytotoxic TNF-α and IL-1/6 protein levels were increased in the AuNR-treated macrophages [62].

#### 3.2.4. Nanomedicine-Based Approaches for Targeting CAFs

CAFs are also key stromal cells responsible for the formation of the immunosuppressive TME in solid tumors because they can recruit immunosuppressive cells and produce a variety of growth factors that promote tumor growth. Furthermore, they create a physical barrier for both drug delivery and tumor infiltration with cytotoxic T cells. To overcome immune evasion mediated by these cells, depletion of this lymphocyte population was considered as a therapeutic possibility. However, according to a recent report, their elimination caused the upregulation of Wnt16, which rendered the neighboring tumor cells resistant to treatment [138]. Hence, the inhibition of tumor CAFs seems to be a more attractive approach. In this context, a novel puerarin nanoemulsion (nanoPue) was developed to downregulate reactive oxygen species (ROS) production in activated CAFs. ROS are actively involved in multiple profibrogenic pathways and are indispensable for CAFs’ activation, and thus, nanoPue demonstrated a strong ability to inactivate CAFs in the TME. As a consequence, collagen deposition in the tumor area was reduced and tumor permeability was enhanced, which improved the chemotherapy effect in the desmoplastic TNBC model, induced a two-fold increase in tumor infiltration with cytotoxic T cells and decreased tumor weight. Moreover, remodeling of TME improved the efficacy of PD-L1 blockade therapy in a TNBC model. Therefore, nanoPue could serve as an adjuvant therapy for chemotherapeutic drugs and immunotherapies in highly desmoplastic tumors, such as TNBC [63].

One different strategy to inhibit CAFs in the TME consists in the injecting a hydrogel to controllably deliver losartan into the tumor area. TGF-β is a key driver of CAFs’ activity as it induces the secretion of collagen I by CAFs, which can stabilize the epithelial-to-mesenchymal transition and promote the invasion of cancer cells and tumor metastasis. Losartan is an angiotensin II receptor antagonist, which inhibits the TGF-β signaling pathway and inactivates CAFs after prolonged and localized administration. Thus, losartan was trapped within a peptide hydrogel network and intratumorally injected in a TNBC murine model. Notably, these polymeric hydrogels have a great potential because they can be easily functionalized, delivered by local injection and degraded into amino acids afterwards. Upon the injection of the hydrogel, Dox-loaded liposomes (Dox-L) were also administered. Losartan hydrogel improved the efficacy of Dox chemotherapy using CAF inactivation, leading to more effective inhibition of tumor growth (64% in comparison with Dox-L alone) and lung metastasis (80% versus Dox-L alone). The described data suggest the high therapeutic potential of this adjuvant therapy [64].

#### 3.2.5. Nanomedicine-Based Approaches for Targeting MDSCs

MDSCs are highly accumulated in tumor sites, where they are able to suppress the activation and proliferation of cytotoxic T cells and stimulate Tregs cells. Hence, nanomedicine-mediated depletion of MDSCs in the TME could be a new perspective in cancer immunotherapy. Examples include a syringeable immunomodulatory multidomain nanogel (iGel), which was designed to both reduce MDSCs in tumors and reprogram the whole TME from a tumor-promoting to a tumor-rejecting immune niche. A positive therapeutic effect of the intratumoral depletion of inhibitory TAMs and MDSC was described in a 4T1 murine model after the application of iGel. This synthetic immune niche was applied as a local postsurgical treatment in the 4T1 murine model to achieve a local and extended release of clodronate, in order to deplete inhibitory TAMs, together with gemcitabine (GEM) and the Toll-like receptor 7 (TLR7) agonist imiquimod (R837), with the aim of triggering the ICD of tumor cells, activating recruited APCs, generating antigen-specific T cells and depleting inhibitory MDSCs at the tumor site. After the treatment with iGel, the TME was characterized by increased levels of infiltrating CD4+ and CD8+ T cells and NK cells, upregulated expression of Th1 cytokines and reduced percentages of MDSCs and M2-like TAMs. In addition, iGel was able to generate a memory T-cell response and inhibit tumor growth and lung metastasis. Finally, the expression levels of PD-1 in T cells and PD-L1 in tumor cells were higher in treated 4T1 tumor-bearing mice. Thus, tumors initially not responding to checkpoint therapy became sensitive to this treatment [65].

A similar implantable synthetic immune niche (iCD) was designed using the MSDCs-depleting agent GEM and a cancer vaccine, composed of whole-tumor lysate antigens together with TLR3 agonists to promote a stronger immune response. The interconnected porous architecture of the iCD provides both local and sustained delivery of drugs and a space for recruited immune cells to interact with nanomedicines and become activated. The iCD acts as an immune-priming center that induces the recruitment and activation of DCs and the proliferation of antigen-specific T cells, reduces the population of MDSCs in the TME and creates a favorable microenvironment for T-cell activity via the local delivery of GEM. Moreover, iCD was successfully used as a postsurgical tumor treatment in an advanced-stage primary 4T1 breast tumor model. It was reported to prevent tumor recurrence at the surgical site, reduce lung metastasis in 60% of the treated mice and achieve a 100% survival rate [66].

In addition, treatment with Dox was suggested to selectively eliminate MDSCs in the spleen and TME and was shown to have immunomodulatory effects [139]. Thus, this chemotherapeutic agent has been proposed to be used as a part of nanomedicines in different types of malignancy, including breast cancer. One of these strategies is a combined administration of a liposomal nanoformulation of HER2/neu-derived P5 peptide together with PEGylated liposomal Dox (Doxil^®^) to treat HER2+ tumor-bearing mice, aiming to inhibit the MDSCs via Doxil^®^ and induce a stronger immune response upon the liposomal P5 immunotherapy. This combination reduced the population and activity of MDSCs in the spleen and TME, while it enhanced the CD4+ and CD8+ T cells’ populations and IFN-γ production. Finally, it decreased tumor size and increased survival rates. Importantly, Doxil^®^ is less cardiotoxic than free Dox and more efficient in MDSC elimination and CTL activation [67].

Cancer treatment with the Dox-polyglycerol-nanodiamond conjugate (Nano-DOX) has been also studied. Nano-DOX displays a lower therapeutic potency than free Dox but exhibits some benefits over the free drug. This nanodiamond was used to treat 4T1 breast tumor-bearing mice and showed better tolerance and less toxicity than free Dox, without causing chemoresistance to Dox in the 4T1 cells, which is a key problem of the free chemotherapeutic agent. In addition, nano-DOX downregulated tumor-derived granulocyte-colony-stimulating factor (G-CSF) and suppressed tumor infiltration with MDSCs and MDSCs’ activation. Finally, it induced the release of DAMPs by 4T1 cells and the subsequent activation of M1 macrophages, DCs and CD4+ and CD8+ T cells in the tumor tissue and stimulation of the antitumor immune response. All these findings together demonstrate that chemotherapeutic drugs in nano-forms acquire new improved properties and these nanomedicines in combination with immunotherapy may provide a more effective treatment of cancer [68].

#### 3.2.6. Nanomedicine-Based Approaches for Targeting Tregs

High-grade tumor infiltration with Tregs is correlated with poor prognosis in breast cancer patients as these lymphocytes inhibit CTLs. Hence, depletion of tumor Tregs is another candidate strategy to potentiate cancer immunotherapy. Ursolic acid (UA)-liposomes have been developed to reduce Tregs within the TME in 4T1 tumor-bearing mice. UA is a potent pentacyclic triterpenoid, found naturally in plants and fruits, which shows immunomodulatory effects due to the inhibition of the JAK/STAT signaling pathways in Tregs. Tregs express high levels of the IL-2 receptor α chain (CD25) that binds to IL-2 and causes the phosphorylation of STAT5, the activation of the JAK/STAT signaling pathway and the subsequent induction of Foxp3 transcription. After the injection of UA-liposomes in a murine breast cancer model, they reached the tumor tissue and reduced IL-10 and IL-6 secretion as well as Foxp3+ phenotypic Tregs cells by inhibiting STAT5 phosphorylation. Despite the fact that UA-liposomes are only able to slow down tumor growth without completely destroying tumor tissue, an in vivo administration of these liposomes led to a depletion of MDSCs and CD4+CD25+Foxp3+ Tregs in the TME [69]. Systemically administered iron-oxide nanoparticles (IONPs) together with PTT also effectively depleted tumor-associated Tregs in a murine 4T1 breast tumor model. Moreover, IONPs-mediated PTT combined with immune checkpoint blockade can significantly inhibit 4T1 primary and distal metastatic tumor growth through the abscopal effect. Remarkably, the combined therapy was able to generate functional memory tumor-antigen-specific T cells to prevent tumor recurrence [70]. In another study, zoledronic acid containing-NPs (NZ) were used to reverse Dox chemoresistance in breast cancer models. NZ inhibited the STAT3/IDO axis and the production of kynurenine, an immunosuppressive catabolite of tryptophan that is produced by IDO. Kynurenine, which is highly expressed on chemoresistant cancer cells, increased the Tregs population and impaired the proliferation and survival of T lymphocytes in the TME. In this study, NZ decreased the number of Tregs and increased the recruitment of DCs in the tumor area, thereby restoring the recognition of resistant tumors via the host immune system. This finding demonstrated that NZ could be used as an adjuvant agent to improve chemo-immunotherapy protocols for chemoresistant breast tumors [71].

Lastly, the novel CAR-T (Chimeric Antigen Receptor T)-cell-mediated cancer therapy is very promising, but its therapeutic effect is poor in solid tumors due to the immunosuppressive TME. To overcome this limitation, lipid NPs were designed to enhance CAR-T-cell therapy in breast cancer. These NPs carried a drug cocktail to simultaneously inhibit immunosuppressive cells within the TME and stimulate antitumor immune cells and they were tested in 4T1 tumor-bearing mice before the infusion of CAR-T cells. In particular, these NPs were loaded with an immunostimulant-invariant natural killer T-cell (iNKT) agonist and a selective inhibitor of the phosphoinositide 3-kinase (PI3K) p110δ isoform, which shows activity against tumor-associated Tregs and MDSCs [72]. Since p110δ is a relevant signaling protein that controls many functions of immune cells’ physiology, the inhibition of PI3K reduces Tregs’ and MDSCs’ proliferation and activity [140]. In this way, prior NPs conditioning reversed the immune-hostile TME and thereby infused CAR-T cells were able to effectively infiltrate the tumor area, undergo robust expansion and eliminate cancer cells. Indeed, preconditioning with these NPs reduced the concentration of TAMs (by 9.4-fold), MDSCs (by 4.6-fold) and Tregs (by 4.8-fold) and increased the density of CD8+ T cells (by 6.2-fold) and iNKT cells (by 29.8-fold) at the tumor site. As a result, NP preconditioning allowed a dramatic accumulation of CAR-T cells in breast tumors (22-fold higher levels) and increased their therapeutic activity. Therefore, this therapy doubled the overall survival when compared to CAR-T-cell therapy alone [72].

### 3.3. Applications of Nanomedicine to Enhance DC Antigen Presentation and Activity

The role of DCs in the antitumor immune response can be directly boosted by two main nanotherapeutic strategies: the induction of the ICD of cancer cells and the subsequent release of intrinsic TAA into the TME or the administration of exogenous TAA into the patients using nanovaccines as vehicles.

#### 3.3.1. Nanotherapies for Inducting the ICD of Cancer Cells

Antigen presentation and DC function are often impaired in the immunosuppressive TME. A promising approach to solve both drawbacks is to increase tumor immunogenicity through the induction of the ICD of malignant cells. ICD stimulates antitumor immunity by different effects on cancer cells, including the promotion of surface expression of calreticulin (CRT) and high-mobility group box 1 (HMGB1) in tumor cells and the increasing of adenosine triphosphate (ATP) secretion. On the one hand, CRT is an ‘eat me’ signal that triggers the phagocytosis of dying tumor cells by DCs, whereas HMGB1 binds to TLR4 and promotes DC maturation and tumor antigen presentation to CTLs. On the other hand, ATP is able to stimulate tumor infiltration with CTLs (Figure 1). Thus, several nanotherapeutic strategies have already been described to elicit ICD in breast tumors. Most of them are based on the application of PDT or PTT to the tumors. For instance, biocompatible Zn-pyrophosphate (ZnP) shell NPs loaded with pyrolipid photosensitizer (PS) in the core can be used to treat 4T1 and TUBO bilateral syngeneic mouse models by PDT [73]. Similarly, a tumor-targeted polypyrrole NP carrying the camptothecin (CPT) chemotherapeutic agent and a near-infrared dye was used to perform synergistic chemotherapy and PTT in 4T1 tumor-bearing mice [74]. Moreover, a polydopamine nanomedicine was simultaneously loaded with carbon dots, a fluorescent agent and the ROS-responsive TLR7/8 agonist resiquimod (R848) to treat 4T1 tumor-bearing mice [75]. These nanosystems were able to induce the ICD of cancer cells and were also combined with PD-L1 blockade therapy. As a result, all of the combined therapies elicited a systemic tumor-specific CTL response. Thereby, they potentiated the effects of PD-L1 inhibition, eradicated both light-irradiated and distant tumors and prevented tumor recurrence and metastasis in mouse models. Notably, combined therapies induced stronger immune response and exhibited higher therapeutic efficacy than immunotherapy alone. In conclusion, nanomedicine-based PDT and PTT can be an effective solution to increase the proportion of cancers responding to immunotherapy [73,74,75].

In addition, several cytotoxic drugs, including oxaliplatin (OXA) and Dox, which have been reported to trigger the ICD of cancer cells, have been encapsulated into nanoplatforms to treat breast cancer. On the one hand, an OXA prodrug and a PEGylated PS have been integrated into a TME-activatable vesicle for treatment in a mouse 4T1 breast tumor model. These vesicles are stable in blood circulation until they arrive at the tumor site, where matrix metalloproteinase-2 (MMP-2) cleaves their PEG corona and the acidic TME triggers the surface charge reversal of these vesicles. It enables tumor-specific penetration, the cellular uptake of vesicles and prodrug activation in tumors. This is followed by laser illumination and the nanoplatform induces ROS generation and drug release, which promotes the ICD of cancer cells. Moreover, αCD47 can be intratumorally injected in mice after the delivery of the vesicles and laser irradiation to block CD47, which is a ‘don’t eat me’ signal overexpressed on the surface of tumor cells that prevent their phagocytosis by DCs. In this way, cancer cells can be phagocytosed after treatment and antitumor immunity is further promoted. Hence, the combination of the nanoplatform with CD47 blockade suppressed tumor growth and distant metastasis and prevented tumor recurrence by triggering a long-term antitumor immunity [76]. In this context, CD47 inhibition could also be improved by a different strategy based on nanomedicine. An acidity-responsive nanocarrier (NP-siCD47/CCL25) was developed to sequentially release siCD47 into cancer cells and CCL25 protein within tumor stroma. CCL25 is a chemokine and the only ligand for CCR9+ T cells. These cells possess an enhanced potential to be activated and produce proinflammatory cytokines and may mediate stronger antitumor response, as they are able to promote CD8+ T cells’ expansion and survival and to inhibit CD4+ T-cells’ differentiation into Tregs. However, CCL25 is not expressed on TNBC cells; therefore, it was proposed to intratumorally deliver CCL25 into a murine TNBC model in order to increase CCR9+CD8+ T-cell infiltration in the tumor. As a result, the promotion of CCR9+CD8+ T-cell infiltration enhanced the antitumor effect induced through the downregulation of CD47 expression and caused tumor growth arrest and the inhibition of metastatic dissemination [77].

In another attempt to induce the ICD of cancer cells in orthotopic 4T1 breast cancer, Dox was encapsulated into highly integrated mesoporous silica NPs (DOX@HIMSNs). DOX@HIMSNs were integrated with a pH and glutathione (GSH) dual-stimulated rotaxane in order to achieve intratumorally specific drug release and were prepared with active targeting and magnetic resonance/computed tomography imaging abilities to increase tumor-positive contrast and guide in vivo therapies. This therapy was able to inhibit tumor growth and metastasis in treated mice [78].

Furthermore, versatile cancer cell membrane (CCM)-coated calcium carbonate NPs carrying low-dose Dox and Ce6 (MC/Dox/Ce6) were developed to release in situ TAAs and serve as a DC vaccine for breast cancer treatment. The CCM is capable of targeting the tumor tissue after being administrated into 4T1 tumor-bearing mice, as it is derived from exogenous cancer cells and exhibits tumor-specific proteins on the surface. Upon laser irradiation, ROS are intracellularly generated and DCs are recruited into the TME. In turn, cancer cells undergo ICD and release TAAs to the TEM, which triggers the maturation of DCs in the tumor area. Hence, MC/Dox/Ce6 promoted a strong antitumor immune response and inhibited both primary and distant tumors after a single administration in combination with PDT. In conclusion, MC/Dox/Ce6 integrates the benefits of chemotherapy, immunotherapy and PDT [80].

Numerous approaches to promote the ICD of cancer cells using different immunoadjuvants have also been designed. Firstly, the polymeric cooper chelator RPTDH was used to assemble pH-sensitive NPs carrying R848 in order to combine immune activation with the antiangiogenesis effect of RPTDH-induced copper deficiency. Since cooper salts (the levels of which are often elevated in breast cancer cells) are a key element in the disease, this nanoformulation dramatically suppressed tumor angiogenesis, proliferation and motility caused by copper inhibition. Furthermore, R848 induced DC maturation and immune activation. Hence, tumor growth and lung metastasis were noticeably inhibited in treated mice [88]. Secondly, NPs characterized by a superior photothermal conversion efficacy (CPCI-NPs) were evaluated as a strategy to carry a PS agent and controllably release R837 adjuvant (CPCI-R837-NPs) into the TME. Synergistic photothermal/immuno-therapy using CPCI-R837-NPs was proven to be considerably effective against breast cancer in a murine model, especially when it was combined with anti-PD-1 antibody treatment. Similarly to the previous results, this therapy was able to eradicate the light-irradiated tumors as well as the light-unexposed ones, through the activation of a systemic antitumor immune response [80]. Moreover, a photothermal agent and R837 were co-encapsulated into poly(lactic-co-glycolic) acid (PLGA) NPs. The formed NPs can be used to apply PTT, induce the ICD of cancer cells and generate TAAs, as it exerts vaccine-like functions together with the immunostimulating effects of R837. These NPs, along with an anti-cytotoxic T-lymphocyte antigen-4 (CTLA-4) antibody, induced a strong antitumor memory immune response in 4T1 breast tumor-bearing mice and were able to eliminate primary and secondary tumors and prevent tumor relapses [81].

Likewise, Dox and R837 were separately encapsulated in low-molecular-weight heparin (LMWH)-d-α-tocopheryl succinate (TOS) micelles (LT). The two types of LT exerted synergistic anti-tumor effects: LT-Dox induced the ICD of cancer cells, while LT-R837 triggered the secretion of different cytokines and activated macrophages, DCs and Th1 immune response in an orthotopic 4T1 breast cancer. In turn, LMWH and TOS micelles were not only drug carriers, but also exhibited strong anti-metastasis effect because they inhibit different phases of the metastatic spread. However, the consequent increase in the cytokine secretion upregulates the expression of immune checkpoints such as PD-L1; thus, the micelles were administered in combination with a PD-L1 inhibitor to eliminate their negative effects. This multifunctional strategy upregulated the maturation of DCs, increased the secretion levels of TNF-α and IFN-*γ*, promoted the Th1 immune response, and increased tumor T-cell infiltration with proper CD8+ CTLs/Tregs and effector CD4+ T cells/Tregs ratios. As a result, the proposed therapy significantly reduced tumor volume and inhibited tumor growth and pulmonary metastasis [82].

Nanotechnological treatment with Dox and R848, which can promote the maturation and activity of DCs, has been evaluated as well. For example, immune nanoconverters (iNCVs) were encapsulated with R848 and Dox and loaded into a designed scaffold. Dox induces the ICD of cancer cells and acts as an in-situ cancer vaccine, while R848 exhibits immunostimulatory effects and can also transform the MDSCs into tumoricidal APCs and repolarizes TAMs to the M1 phenotype. This nanoplatform promotes immunogenic phenotypes in tumors, enhances systemic and long-term antitumor immune response, transforms α-PD-L1/α-PD-1-nonresponsive tumors into responsive tumors and prevents tumor recurrence and metastasis in a 4T1 breast cancer model [83]. In addition, a dual pH-responsive NP system was designed by the encapsulation of R848 with poly(L-histidine) (PHIS) to form nanocores, which were coated with prodrug hyaluronic acid (HA)-Dox. HA-Dox/PHIS/R848 NPs specifically released Dox and R848 into the acidic TME of 4T1 tumor-bearing mice and dramatically inhibited the tumor growth by potentiating the antitumor immune response [84].

Oligodeoxynucleotides containing cytosine-guanine motifs (CpG-ODN) are TLR9 agonists that have been evaluated for their application in cancer nanotherapy as immunoadjuvants. NPs composed of a polymeric core and a gold NP-based coat were used to deliver oligodeoxynucleotides containing cytosine-guanine motifs (CpG-ODN), TLR9 agonists, together with zinc phthalocyanine (ZnPc) PS into mouse 4T1 breast cancer cells. Notably, the CpG-ODN were uptaken into TLR9-rich endosomes of plasmacytoid DCs and they specifically activated TLR9 signaling and induced the activation of DCs. Preliminary results demonstrated the benefits of combining the phototoxic and immune-stimulating activities of PDT with the enhancement of DC activation by CpG-ODN to deal with breast cancer [86]. Furthermore, CpG-ODN were loaded into light-responsive chitosan-coated hollow CuS NPs and these NPs were combined with PTT in a mouse breast cancer model. The combined therapy has been proved to efficiently induce the ICD of malignant cells and potentiate the immune response against cancer cells. It reduced primary and distant tumor growth more significantly than PTT or immunotherapy therapy alone [87].

Lastly, another described strategy to induce the ICD of breast cancer cells by nanotherapy is based on a tumor-specific enhanced oxidative stress polymer conjugate (TSEOP). Under high concentrations of H_2_O_2_ and low levels of pH in the TME, TSEOP specifically releases quinone methide (QM) and generates cinnamaldehyde (CA). QM acts as a GSH scavenger, while CA is a ROS amplifier. In this way, through the cooperative depletion of GSH and the generation of ROS, TSEOP is able to induce strong oxidative stress in tumor cells. It subsequently leads to endoplasmic reticulum stress responses and the ICD of cancer cells is induced. TSEOP, characterized by great tumor selectivity, potentiated the maturation of DCs, boosted antitumor-specific immunity and completely eradicated breast tumors in treated mice [85].

#### 3.3.2. Peptide-Based Nanovaccines

The administration of cancer vaccines is an alternative approach to enhance tumor antigens’ presentation to APCs. Peptide-based vaccines are the most common vaccines that have been designed for breast cancer. They enable DCs to present TAAs T cells and elicit antigen-specific immune responses. Several promising peptide vaccines are under ongoing clinical trials in patients with breast cancer, but still with limited efficacy. For that reason, nanocarriers are beginning to be used as cancer vaccines, as they are able to specifically deliver various antigens with improved stability and act as potent immunoadjuvants. Currently, different types of peptide-based nanovaccines are being evaluated for breast cancer therapy, such as VLP-based, liposomal or polymeric vaccines. Additionally, the repertoire of TAA administered in nanovaccines for breast cancer is constantly expanding.

VLPs have been proposed by many studies as immunogenic adjuvants to enhance the potency of peptide vaccines, since their viral RNA is a natural ligand for TLR7 and their protein structures are also immunogenic. A phase I clinical trial, which offers promising preliminary results, was developed to use an anti-HER2/neu vaccine in patients with metastatic breast cancer (MBC). HER2/neu is a protein that is overexpressed in 15–20% of breast cancers, and is thought to promote metastasis and disease progression. Aiming to increase the antigenicity of the self-antigens, three B-cell epitopes derived from the extracellular domain of the HER2/neu protein were coupled to immune-potentiating reconstituted influenza virosomes (IRIV). After three intramuscularly applications of the virosomal formulated vaccine, it elicited specific B- and T-cell immune responses in 8 of 10 patients. In these eight patients, peptide-specific antibodies for all the epitopes were generated and the production of IL-2, IFN-γ and TNF-α was increased, whereas the number of CD4+CD25+Foxp3+Tregs was reduced [89].

Influenza VLPs have been also used to deliver HER2 in a glycosylphosphatidylinositol (GPI)-anchored form into breast tumor-bearing mice. After vaccination, strong Th1- and Th2-type immune responses were promoted and the HER2-specific IgG production was enhanced, while the soluble form of GPI-HER2 weakly induced the Th2 response. Furthermore, vaccinated mice were protected against challenge with HER2-expressing tumor [90]. Moreover, VLPs with a high-density surface display of the HER2 extracellular domain were demonstrated to stimulate therapeutically potent and durable anti-HER2 CTL-based responses in a murine model. As a result, these VLPs prolonged the survival of treated mice and prevented tumor growth as well as spontaneous tumor development [98]. Additionally, a heterologous prime-boost strategy based on the presentation of the unique HER2 B-cell epitope (CH401) by three different VLPs was evaluated to treat HER2+ breast cancer. The three VLPs were based on CPMV, cowpea chlorotic mottle virus (CCMV) and Sesbania mosaic virus (SeMV). Each nanovaccine was sequentially administered in vivo only once in order to focus the immune responses on the epitopes. This regimen of vaccination elicited higher titers of CH401-specific immunoglobulins, stronger Th1-predominant response and more cytotoxicity towards cancer cells than a repeated vaccination regimen. The heterologous prime-boost regimen reduced tumor growth and enhanced survival in treated mice more significantly than traditional vaccination, which proves that novel vaccination strategies could improve their efficacy against cancer [99].

Finally, VPLs were proposed as an approach to inhibit cancer stem cells (CSC). CSC are related to tumor relapse, metastasis and therapeutic resistance and they are dramatically abundant in aggressive forms of breast cancer. With this purpose, a VPL-based vaccine (AX08-0M6) was designed using a family of RNA bacteriophages to display on its surface an extracellular domain of human cystine-glutamate antiporter protein xCT, a key element in CSC function that is highly expressed in breast tumors. AX08-0M6 was proven to effectively impact CSC biology via the production of high levels of anti-xCT IgG2. Thus, it inhibited tumor growth and pulmonary metastases in preclinical breast cancer models [100].

In addition, liposomal NPs carrying different peptides have also been reported to potentiate the efficacy of vaccines for breast cancer. For instance, multi-epitope P5 peptide was encapsulated into nanoliposomes composed of 1,2-dioleoyl-3-trimethylammonium propane (DOTAP), which stimulates DCs, cholesterol (Chol) and Polyriboinosinic: polyribocytidylic acid (Poly (I:C)), which is a strong immunoadjuvant that enhances Th1 and CTL responses. The formulation was administered to TUBO tumor-bearing mice three times at two-week intervals. Nanoliposomes carrying P5 were introduced into APCs’ cytosol due to their cationic liposomal composition. As a result, this nanoformulation enhanced the release of IFN-γ by CD8+ T lymphocytes, reduced the tumor growth rate and provided protection against tumor regression in treated mice [101]. In the same regard, dioleoyl-phosphatidyl ethanolamine (DOPE) was used to design different liposomes carrying P5 in order to release the peptide into the cytosol of APCs, especially in DCs. These liposomes also delivered monophosphoryl lipid A (MPL), which stimulates TLR4 and induces the production of co-stimulatory molecules and inflammatory cytokines via DCs. As a result, the presentation of P5 to CD8+ lymphocytes through APCs was enhanced. The Lip-DOPE-P5-MPL formulation was subcutaneously injected three times into a TUBO tumor mice model and it induced significant CTL response against the P5 antigen and increased IFN-γ secretion by CD8+ T cells. Lip-DOPE-P5-MPL vaccination inhibited tumor growth and extended survival time in treated mice, as the combination of MPL and DOPE has synergistic effects on boosting vaccine efficacy [102]. Similarly, the same DOPE liposomal formulation containing MPL and Pan HLA-DR peptide (PADRE) was used to deliver P5 peptide [103] or long multi-epitope HER2/neu-derived E75-AE36 (linkage of two short peptides) [104] into TUBO-bearing mice. The addition of PADRE to liposomes activated a powerful antitumor response based on CD4+ T helper cells and remarkably enhanced immune responses against P5 and E75-AE36 peptides, when compared to the free peptides or the same liposomal formulations without PADRE [103,104]. In addition, synthetic long peptides are able to induce stronger long-lived immune responses than short peptides because the synthetic long peptide contains optimum CTLs and Th epitopes. E75 binds to HLA-A2 and HLA-A3 molecules and stimulates CTLs, while AE36 binds to MHC class II molecules and potentiates CD4+ Th and CD8+ T cells [103,104].

Short E75 and AE36 peptides encapsulated into different nanoliposomes have also been used in combination with other adjuvants. The AE36 peptide was incorporated into nanoliposomes composed of DOTAP, DOPE and cholesterol (DDC) or only DD, together with the CpG motif. After the injection of these liposomal nanoformulations into a TUBO breast tumor murine model, increased IL-4 and IFN-γ secretion, reduced tumor size and prolonged survival time in therapeutic and prophylactic models were observed [105]. E75 was incorporated into liposomes composed of phospholipids, cholesterol and DOPE and similarly promising results were obtained in TUBO tumor-bearing mice [91]. Furthermore, a different liposomal formulation of E75 peptide (Lip-Pep) was injected three times into TUBO tumor-bearing mice, in combination with three injections of a liposomal formulation of Dox (Lip-Dox). The treatment with Lip-Dox and Lip-Pep promoted tumor infiltration with TILs and NK cells, stimulated IFN-γ secretion and reduced MDSCs and CD25+Foxp3+ Tregs populations in the TME more efficiently than E75 and Dox alone. Notably, mice treated with Lip-Pep+Lip-Dox showed a more significant reduction in tumor growth rates and the highest survival time [92].

Besides, Gp2 peptide (derived from the transmembrane domain of HER2/neu protein) was conjugated to micelles and then inserted into liposomes composed of DOPE, which contained MPL. This formulation (Lip-DOPE-MPL-GP2), used to immunize TUBO tumor-bearing mice, stimulated IFN-γ production by CD8+ T cells as well as antigen-specific CTL responses and it led to a reduction in tumor size and an increasing of the survival time of treated mice [93].

In addition, polymeric NPs carrying different peptides were used as nanovaccines for breast cancer. Firstly, polymeric NP, which combines MHC-I and MHC-II HER2 peptides with CpG and MPL (EntrapNP), was tested in an orthotopic HER2+ breast cancer model as a nanovaccine. EntrapNP was efficiently internalized by DCs and the exogenous antigens were cross-presented through MHC pathways. After three doses of the nanovaccine, tumor infiltration with TILs (mainly cytotoxic memory-T cells) was increased and an antigen-specific immune response against cancer cells was triggered. Treated mice showed a significant delay in tumor growth and lower incidence of metastatic lesions [94]. Secondly, PLGA NPs containing the designed CpG-coated tumor antigen (Tag) were shown to be avidly endocytosed and presented by APCs, especially by DCs. The encapsulated tumor antigen was composed of a membrane lysate of 4T1 tumor cells. As a result, CpG-NP-Tag NPs increased the DC maturation and activation status, stimulated the tumor-specific CTL response and attenuated breast tumor growth and angiogenesis in vivo [95]. In another study, PLGA-NPs were used to encapsulate Hp91, an immunostimulatory peptide derived from HMGB1. Studies in vitro demonstrated the ability of this nanovaccine to robustly activate DCs, as compared to the free peptide. Moreover, vaccination of a murine breast cancer model with PLGA-Hp91-NPs together with HER2 free peptide enhanced the activation of HER2-specific CTLs’ response, inhibited tumor development and prolonged survival time more significantly than HER2 peptide alone due to the immunoadjuvant potency of NPs [96].

Finally, the co-localized delivery of a nanomedicine and an antigen epitope-based nanovaccine was proposed to directly program DCs in vivo. An injectable and thermosensitive hydrogel carrying curcumin-loaded polymeric NPs (nanomedicine) and a nanovaccine was developed to completely cover the surgical bed of primary tumors and treat residual cancer cells after surgery through the sustained delivery of nanotherapy. On the one hand, curcumin NPs are able to both stimulate the recruitment and the maturation of DCs and enhance tumor immunogenicity by inducing the ICD of the residual tumor cells. On the other hand, in order to amplify the antitumor T-cell response, E75 peptide and CpG-ODN were co-assembled in a cationic polymeric NP to act as a nanovaccine. Thus, after insertion of the hydrogel in the postoperative 4T1 breast carcinoma model, the infiltration of CD8+ T cells in the relapsed tumor increased, the systemic antitumor immune response was synergistically triggered and tumor recurrence and pulmonary metastasis were significantly attenuated in treated mice [97].

#### 3.3.3. Gene-Based Nanovaccines

Nanomedicine has been also used to improve gene-based vaccines. Several DNA and RNA-based nanovaccines are currently being evaluated as therapies for breast cancer. For instance, the delivery of a xenogeneic telomerase reverse transcriptase (*TERT*) DNA vaccine after the intramuscular administration of chemokine ligand 21 (*CCL21*) was studied. On the one hand, human *TERT* (*hTERT)* regulates cell proliferation and immortality and it is a potential TAA for immunotherapy since it is overexpressed by cancer cells. Thus, the COOH terminal part of the *hTERT* gene (*cTERT*) was fused with the *PADRE* sequence and a ubiquitin sequence to enhance protein presentation. The resulting DNA vector was encapsulated into liposomes with hemagglutinating virus of Japan coating. On the other hand, *CCL21* is secreted in lymph nodes and contributes to the recruitment of naïve T cells and *CCR7*-expressing mature DCs. Hence, *CCL21* injection activated DCs at the vaccination site and elicited a stronger antitumor immune response against *TERT*-expressing cancer cells. The combined therapy, in prophylactic and therapeutic models of *TERT*-expressing breast cancer, induced stronger antitumor responses based on anti-*TERT* specific CTLs, triggered the Th1 immune response more predominantly, and inhibited tumor growth more strongly as compared to the *cTERT* DNA vaccine alone [106].

Regarding the route of administration, oral delivery of DNA-based vaccines is a noninvasive method that has some advantages over intravenous vaccination. Alginic acid-coated chitosan NPs (A.C.NPs) were evaluated as an oral carrier for the legumain DNA vaccine in a murine orthotopic 4T1 breast cancer model due to their excellent stability, biocompatibility and capacity to enhance mucosa absorption of the drugs. A.C.NPs showed higher efficacy than the oral DNA vaccines, as they were able to resist DNA degradation in the acidic gastric environment and they increased DNA uptake and expression by APCs in the intestinal Peyer’s patches. Furthermore, as legumain is an asparaginyl endopeptidase that is overexpressed on TAMs, this oral vaccination system increased the amount of activated CTLs targeting TAMs and remodeled the immunosuppressive TME. Therefore, A.C.NP treatment inhibited activated Tregs cells in the TME, suppressed tumor growth and prolonged the survival of treated mice [107].

RNA-based vaccines are also being evaluated to treat breast cancer due to the two major advantages of this modality over DNA vaccines. Firstly, RNA-based vaccines can be delivered into the cytoplasm of targeted cells to produce the proteins of interest, whereas DNA-based vaccines have to integrated into the cellular nucleus to be transcribed into mRNA that will be later translated into the proteins of interest in the cytoplasm of targeted cells. Thus, RNA-based vaccines are able to produce higher levels of protein in a shorter period of time. Importantly, RNA-based vaccines are safer to use than DNA-based vaccines, since they do not integrate a foreign DNA in the genome of targeted cells. For that reason, lipid/calcium/phosphate NPs modified with mannose were evaluated to deliver an mRNA vaccine into the cytoplasm of DCs. The mRNA encoded MUC1 tumor antigen, a mucin that is overexpressed in breast carcinoma cells. The application of these NPs in combination with the anti-CTLA-A antibody into an orthotopic TNBC model induced a stronger antigen-specific CTL response against cancer cells and higher IFN-γ production, as well as a more significant tumor inhibition than monotherapies alone [108].

Notably, conventional DC vaccines are not therapeutically efficient in breast cancer because of the immunosuppressive TME. In order to improve the antitumor effect of DC vaccines, a strategy based on nanomedicine has been evaluated in the 4T1 murine model. The first step consisted in the intravenously administration of CD73-specific siRNA-loaded chitosan-lactate NPs to attenuate the immunosuppressive TME. CD73 is overexpressed in cancer cells and exerts immunosuppressive effects through the production of high levels of adenosine. Thus, CD73-specific siRNA-loaded NPs were able to decrease CD73 expression in tumor cells and inhibit adenosine production in the tumor site, which downregulated Treg and MDSC populations, as well as IL-10 secretion. Then, a tumor lysate pulsed DC vaccine was intradermally injected close to the tumor area. In this combined therapy, the efficacy of the DC-based vaccine was potentiated, leading to the activation of the CTL antitumor function, the stimulation of T-cell proliferation and the enhanced production of IFN-γ and Il-17. This treatment protocol inhibited tumor growth, prevented lung metastasis by attenuating the expression and activity of MMPs 2 and 9, and significantly increased survival time in treated mice [109].

### 3.4. Nanomedicine-Based Approaches for Promoting Antitumor T-Cell Response

**Figure 2:**
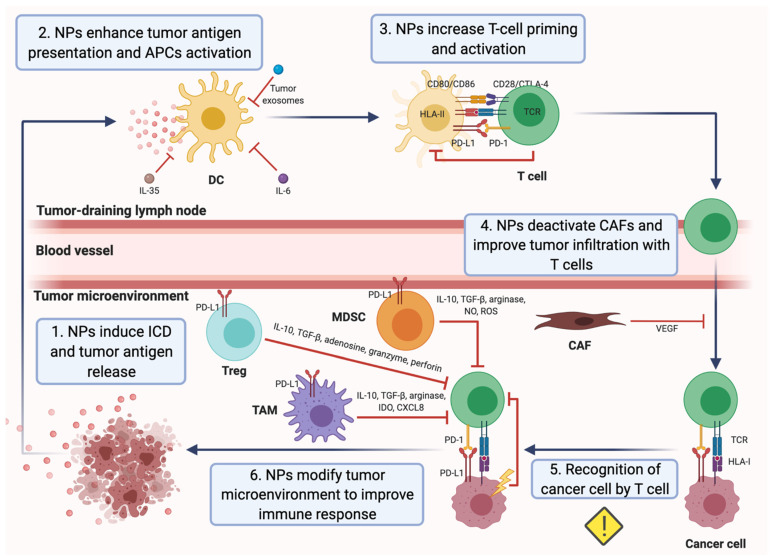
Cancer immunity cycle and nanoparticle (NP)-based strategies to overcome mechanisms of immune escape. NP-based approaches can ameliorate various mechanisms of immune evasion in order to finally induce a strong antitumor T-cell response that inhibits tumor growth in breast cancer. (1) NPs loaded with an extensive repertoire of drugs have been demonstrated to induce the immunogenic cell death (ICD) of cancer cells in breast tumors. (2) NPs are able to enhance the presentation of the released tumor antigens by dendritic cells (DCs), as well as the maturation and activation of DCs, hampered in the tumor microenvironment (TME). (3) Upon the interaction between HLA-II-presenting tumor antigens on DCs and the T-cell receptor (TCR), T cells are activated. Thus, NPs increase T-cell priming. Furthermore, NPs can prevent T-cell inactivation by DCs through the blockade of PD-1/PD-L1 and CD86-CD80/CTLA-4 interactions. (4) Carcinoma-associated fibroblasts (CAFs) form a physical barrier in the TME and inhibit the function of T cells via the secretion of the vascular endothelial growth factor (VEGF). NPs deactivate CAFs and improve the tumor infiltration of specific effector T cells. (5) The infiltrated T cells specifically recognize cancer cells via TCR and tumor HLA-I/peptide interaction. Nevertheless, cancer cells often exhibit alterations in the expression of the HLA-I, which complicates their recognition. Despite that, there is not any NP-based strategy to overcome this mechanism of immune escape in breast cancer yet. (6) Tumor cells and immunosuppressive cells within the TME can hinder the activation of T cells or promote their inactivation. Several NP-based approaches consist in inactivating or reducing immunosuppressive myeloid dendritic stem cells (MDSC), regulatory T cells (Tregs) and tumor-associated macrophages (TAMs) in the tumor area to improve the antitumor T-cell response. Similarly, the inhibition of the indoleamine 2,3-dioxygenase (IDO), IL-10 production and PD-1/PD-L1 interaction promotes the activity of the effector T cells.

Most of the previously mentioned nanomedicines exert indirect effects on the antitumor response based on T-cell responses. However, some nanotechnological approaches have been designed in order to directly potentiate antitumor effector T cells in breast cancer by enhancing CTL activation in the TME, blocking CTL inactivation by tumor cells or promoting the antitumor Th1 response and diminishing the Th2 response (Figure 2).

#### 3.4.1. Nanotherapies for Promoting CTL Activation

CTL activation is often impaired in the immunosuppressive TME. Thus, cancer immunotherapy using genetically modified exosomes has been proposed to promote the activation of the antitumor CTL response. Exosomes have some unique therapeutic advantages over conventional NPs: they exhibit high biocompatibility, low immunogenicity and abundant membrane proteins, such as CD9, which promotes the membrane fusion of exosomes with target cells and enables direct cytosolic delivery of therapeutic drugs. Another membrane marker, CD47, protects exosomes from phagocytosis. A novel exosome platform, named as synthetic multivalent antibodies retargeted exosomes (SMART-Exos), was designed to express on the surface two distinct monoclonal antibodies: anti-human CD3 and anti-human HER2 antibodies. SMART-Exos are able to enhance the lateral stabilization of the clustering between HER2 antigens and CD3 molecules of T-cell receptors. Thus, they specifically recruit CTLs to the TME and facilitate their activation towards the HER2+ breast cancer cells. This nanoplatform exhibited a potent anti-tumor activity both in vitro and in vivo studies and inhibited significantly tumor growth in treated mice [110].

Similarly, the administration of DC-derived exosomes (DCs-Exo) was proposed as a novel cancer immunotherapy tool. DCs-Exo transport many of the immune function-associated molecules of DCs and can be assimilated by different cell types, including tumor cells and T cells. Considering it, DCs-Exo were used to treat human breast adenocarcinoma cells (SK-BR-3) and to stimulate previously SK-BR-3-primed CD3+ T cells. DCs-Exo were incorporated into tumor cells and increased in them the expression of molecules involved in the antigen presentation. Thus, the ability of cancer cells to activate T cells was potentiated. Furthermore, immature DCs in the TME can also capture DCs-Exo and acquire a mature phenotype. In addition, DCs-Exo-treated cancer cells were able to induce previously sensitized T cells to secrete higher levels of IFN-γ than non-DCs-Exo-treated cancer cells. In conclusion, DCs-Exo may act as a tool to enhance the immunogenicity of tumor cells and the efficacy of any cancer immunotherapy strategy [111].

#### 3.4.2. Nanotherapies for Inhibiting IDO-1

Since hampering CTL inhibition in the TME is an efficient approach to potentiate the antitumor T-cell response, several nanomedicines have been rationally designed to block the immune checkpoint of T cells, IDO-1 and PD-1/PD-L1 in particular.

Although PDT and PTT have a potential to elicit a robust antitumor immune response, they increase the infiltration of IFN-γ-secreting cells into the TME and IFN-γ upregulates the expression of PD-L1 and IDO-1 by tumor and tumor-associated immune cells. This mechanism maintains the immunosuppression within the TME and reduces the clinical efficacy of these therapies. Therefore, vesicles were designed in order to overcome the IDO-1-mediated immune escape and strengthen the PDT-triggered antitumor immune response. These vesicles carried a PS and a GSH-sensitive prodrug of the NLG919 IDO-1 inhibitor. Upon the intravenous injection of the vesicles into subcutaneous 4T1 tumor-bearing mice, they accumulated within the tumor area through the MMP-2-mediated cleavage of their corona and NLG919 was specifically released here. This combination of PDT and IDO-1 blockade had synergistic effects and the therapeutic efficacy of the combined therapy was higher than the efficacy of the PDT alone. In this way, PDT with prodrug vesicles dramatically inhibited the tumor growth, suppressed the tumor recurrence and prolonged the survival time of the treated mice [112]. Likewise, PTT has been proposed in combination with the inhibition of IDO-1 by different nanomedicine-based approaches. Micelles carrying a PS and NLG919 were demonstrated to effectively enhance the antitumor immune response induced by PTT. As a result, they not only suppressed the growth of primary breast tumors but also alleviated the lung metastasis of 4T1 tumors in mice [113].

Nanomedicine-mediated PDT together with the inhibition of IDO-1 can be also combined with chemotherapy. For instance, a macrophage membrane-coated shape changeable nanomedicine was designed to deliver Ce6, the GSH-sensitive IDO-1 inhibitor indoximod (IND) and ROS-activatable dimeric paclitaxel (PTX) into breast tumors. The use of the covering macrophage membrane improves drug stability in blood circulation, tumor targeting, intratumoral distribution and cellular internalization via membrane proteins of the micelles. Furthermore, the Ce6-based PDT generates ROS, which induces the change of the spherical micelles into nanofibers. The shape-changeable properties of this nanosystem have certain advantages: spherical micelles provide good blood circulation of the drugs, whereas linear nanofibers allow a strong retention of nanocarriers in the tumor area and the release of drugs on site. In turn, ROS are able to directly kill tumor cells and stimulate the production of monomeric PTX, which can induce the ICD of tumor cells. This multimodal therapy strongly suppressed the growth of breast cancer tumors and inhibited lung metastasis in a mouse breast tumor model, as a result of the synergistic strengthening of antitumor immune activity [114]. Moreover, a light-inducible nanocargo (LINC) was developed for cancer immunotherapy. LINC was composed of a GSH-responsive PS, NLG919 and a light-activatable prodrug of OXA. After the intravenous injection of LINC into 4T1 tumor-bearing mice, the first-wave of near-infrared (NIR) laser irradiation induced the cleavage of the LINC corona and the cellular uptake of the PS, NLG919 and OXA, which were restored by tumor GSH. Then, a second-wave of NIR laser irradiation was applied as PDT to induce the ICD of tumor cells. Thus, LINC inhibited the tumor growth, lung metastasis and tumor recurrence, while it reduced the systemic side effects of chemotherapy [115]. In the same way, OXA was combined with NLG919 using a tumor-microenvironment-activatable binary cooperative prodrug (BCPN) to treat 4T1 tumor-bearing mice. BCPN was designed using the tumor acidity and GSH dual-activatable OXA prodrug and a GSH-responsive homodimer of NLG919. Both drugs are specifically activated in the tumor cells and synergistically modulate the antitumor immune response. This nanosystem showed significantly higher efficiency than free OXA together with NLG919 in inhibiting tumor growth and preventing metastasis in vivo [116].

Lastly, Dox and PTX have also been evaluated in combination with IDO-1 inhibitor to treat breast cancer. A liposomal nanocarrier was constructed by the self-assembly of a phospholipid-conjugated IND, followed by the loading of Dox. Dox/IND-liposome was intravenously injected in an orthotopic 4T1 tumor model and promoted a synergistic antitumor response. Notably, this nanocarrier dramatically improved the pharmacokinetics, tumor concentrations and safety of drugs. Furthermore, the combination of Dox/IND-liposome with an anti-PD-1 antibody eradicated lung metastasis. This fact demonstrated that the antitumor immune response can be triggered through the induction of the ICD of cancer cells and boosted by the blockade of immune checkpoint [117]. In addition, two immunostimulatory polymeric nanomicelles were proposed to specifically deliver IND together with Dox into breast tumors. Both strategies enhanced the antitumor immune response and improved clinical outcomes in orthotopic murine breast cancer models [118,119]. Furthermore, a similar dual-functional prodrug nanomicellar carrier was developed to deliver NLG919 and PTX into breast tumors and it offered similarly encouraging results in a breast cancer mouse model [120].

#### 3.4.3. Nanotherapies for Blocking PD-1/PD-L1

The inhibition of the PD-1/PD-L1 immune checkpoint by nanomedicine has also received attention and interest in recent years. Indeed, nanosystems based on the blockade of PD-1/PD-L1 have been associated with different therapies to increase their efficacy. Firstly, the inhibition of PD-1/PD-L1 was combined with nanomedicine-mediated PTT. The NPs used were composed of photothermal materials (polydopamine NPs) and acted as a biodegradable and adaptable nanoplatform to induce PTT, while they delivered a PD-L1 inhibitor (JQ1) into TNBC tumors. JQ1 is a bromodomain and extra-terminal (BET) inhibitor that is able to downregulate the expression of PD-L1 in TNBC cells by reducing the transcription of the TNBC-promoting gene c-MYC. Simultaneously, JQ1 specifically suppresses the BRD4-c-MYC axis in TNBC cells and induces the apoptosis of these cells. Thus, this JQ1 delivery nanoplatform can be used to combine PTT, immunotherapy and c-MYC-targeted therapy for the treatment of TNBC. In this way, the JQ1-loaded polydopamine NPs (PDMN-JQ1) were able to promote the activation of CTLs and induce a strong antitumor immune-memory response towards primary or distant breast tumors. Thus, they contribute to the suppression of tumor growth and the prevention of tumor recurrence in vivo. After a single intratumoral injection of NPs, JQ1 was continuously released for up to two weeks and the synergistic therapeutic effect of PDMN-JQ1 was notably greater than the effect of free JQ1 and PDMN treatments against TNBC [121]. NPs were also designed to deliver Ce6 and a small-molecule inhibitor of the PD-1/PD-L1 interaction, BM-202, into 4T1 tumor-bearing mice. This nanosystem together with PTT was demonstrated to possess therapeutic effects in breast cancer, mimicking those of conventional anti-PD-L1 monoclonal antibodies. Thus, the inhibition of tumor growth and metastasis caused by these NPs was similarly promising [122]. In addition, PDT and PD-1/PD-L1 blockade were also combined with chemotherapy through the development of hyaluronidase-triggered size-reducible biomimetic NPs (mCAuNCs@HA). These NPs were co-loaded with a PS, a ROS-responsive PTX dimer prodrug and an anti-PD-L1 peptide (dPPA). With the aim of increasing the time in blood circulation and tumor targeting of drugs in 4T1 tumor-bearing mice, NPs were coated with a red blood cell membrane. The triple therapy-activated tumor-infiltrating CTLs and NK cells and increased the production of TNF-α and IL-12, resulting in a significant inhibition of tumor growth and a total absence of lung metastasis in vivo [123].

In a different approach, immune checkpoint blockade was combined with chemotherapy using a MMPs/acidity dual-sensitive NP with a micelle-liposome double-layer structure (PM@THL). NP was loaded with PTX, the anti-CSC agent thioridazine (THZ) and a PD-1/PD-L1 inhibitor (HY). At the TME, MMPs triggered the dissociation of the outer layer of NP and HY, THZ and micelles with PTX were released. The intracellular drugs’ disposition suggests that HY would enter into tumor cells via diffusion, while THZ would be up-taken by tumor cells upon the interaction with PD-L1 on the surface of cancer cells and PTX micelles, then they would be internalized by endocytosis releasing PTX inside them under the endo/lysosome acidity. Hence, PM@THL showed the previously mentioned advantages of changeable-size NPs, while they remarkably reduced the proportion of CSC and increased the tumor infiltration with CTLs. Therefore, PM@THL exhibited an excellent antitumor efficacy and prolonged the lifespan in an orthotopic MCF-7 metastatic breast cancer model [124].

Moreover, the inhibition of PD-1/PD-L1 by nanomedicine has been combined with the administration of the antitumor necrosis factor receptor superfamily member 4 (aOX40). aOX40 is an agonistic antibody which activates costimulatory receptors and induces T-cell activation. The spatiotemporally controlled co-administration of anti-PD-1 antibody together with aOX40, using dual immunotherapy NPs (DINP), was evaluated as a strategy to treat breast cancer. DINP promoted synergistic pro-activation signaling of T cells; thus, it elicited higher rates of T-cell activation and T-cell cytotoxicity in vitro and in vivo than free therapeutic antibodies or single NPs. This approach resulted in enhanced therapeutic efficacy and increased antitumor immunological memory [125].

Finally, a peptide nanodelivery system was proposed to co-deliver a siPD-L1 and an IDO-1 inhibitor as a strategy to block both immune checkpoints. The nanosystem specifically accumulated in the breast tumors because it is based on breast cancer homing and penetrating peptides and locally releases both immune checkpoint blockers in vivo. Notably, siPD-L1 was able to specifically suppress the de novo synthesis of PD-L1 in the TME and block the interaction between PD-L1 and PD-1. The synergistic effects of PD-L1 and IDO-1 inhibition resulted in a greater activation of tumor-infiltrating CTLs, an increase in tumor cell apoptosis and a higher survival time in treated mice, when compared with free siPD-L1 or the IDO-1 inhibitor [126].

#### 3.4.4. Nanotherapies for Promoting Antitumor Th1-Type Response

Numerous nanomedicines have been developed to potentiate the pro-inflammatory antitumor Th1-type immune response and minimize the anti-inflammatory Th2-type response in breast cancer. Selenium (Se) NPs were demonstrated to act as nanovaccines in 4T1 tumor-bearing mice to trigger an antitumor Th1 immune response. Se is an essential micronutrient element which exhibits antioxidant and anticarcinogenic properties as well as cancer-preventative effects and immunomodulatory activity. Indeed, dietary Se deficiency has been associated with immunosuppression and higher incidence of different cancers, while the administration of Se-ion dietary supplements has been demonstrated to inhibit several tumors in murine models and to restore immunologic functions. In this regard, several studies are now directed towards SeNPs due to their excellent biological properties, which are similar to those of Se ions at even lower doses, together with their lower toxicity [127]. In all of these novel strategies, SeNPs were produced by lactic acid bacteria (LAB) since they can reduce Se ions to elemental biogenic SeNPs and accumulate them into intracellular spaces. For instance, biogenic SeNPs were extracted from *Lactobacillus plantarum*, a friendly intestinal bacterium, and orally administered to 4T1 breast tumor-bearing mice. After the administration, the levels of pro-inflammatory cytokines were increased, which indicates that SeNPs promote the Th1-type immune response [128]. Furthermore, whole LAB containing SeNPs have been used due to the beneficial effects of these bacteria on the host immune system. LAB are able to increase NK cells’ activity, optimize the immune system and promote pro-inflammatory responses. Therefore, these NPs would have the simultaneous benefits of Se and probiotics. SeNPs-enriched *Lactobacillus plantarum* [129] and SeNPs-enriched *Lactobacillus brevis* [130] were orally administered to 4T1 tumor-bearing mice and both types increased the production of Th1 cytokines and raised the NK cells’ activity in the treated mice. Furthermore, SeNPs-enriched *Lactobacillus brevis* decreased the level of liver metastasis. In the three previous studies, the rate of tumor growth dramatically decreased as a result of the promotion of the Th1 immune response and cellular immunity. Moreover, after oral administration of the three different SeNPs, the survival rate of tumor-bearing mice was increased up to a 30-day, 130-day and 230-day period, respectively [128,129,130]. In addition, oral supplementation of SeNPs that were produced by *Lactobacillus brevis* together with the injection of crude antigens derived from 4T1 cells was administered to breast tumor-bearing mice. The antitumor Th1 immune response was enhanced and similarly promising results were obtained [131].

Different metal-based NPs have been also proposed to elicit the antitumor Th1 response in breast cancer. Nano-metalloids NPs are considered efficient nanocarriers for the delivery of tumor antigens into APCs in the lymph nodes. For example, chitosan-coated green synthesized copper oxide NPs (CuONPs@CS) have been demonstrated to play an immunogenic role and induce the activation of the antitumor immune response. Indeed, CuONPs@CS carrying tumor lysate antigens were able to activate macrophages after being internalized by them. In turn, activated macrophages induced the activation of antigen-specific Th1 and Th2 cells, enhanced the secretion of pro-inflammatory cytokines and increased the CD4+ T cells’ population, as evidenced by the inhibition of breast cancer proliferation in vitro as well as in vivo and the prolongation of the survival of tumor-bearing mice. Remarkably, the treatment with CuONPs@CS induced Th1 polarization due to the increased production of IFN-γ and IL-12, which promoted the Th1 response and inhibited Th2 cytokines [132].

Lastly, the intratumoral insertion of a nanofluidic-based drug eluting seed (NDES) is a minimally invasive method to provide sustained local delivery of OX40 and CD40 monoclonal antibodies into breast tumors and to avoid repeated injections in the tumor area. On the one hand, CD40 is a costimulatory protein expressed by APCs; thus, its activation is important for the maturation of APCs and ultimately leads to the Th1 response polarization and the priming of CTLs. Moreover, CD40 is also expressed by some tumors and its activation can lead to cancer-cell apoptosis. On the other hand, OX40 is a co-stimulatory molecule expressed by activated CD4+ and CD8+ T cells, which enhances the proliferation, survival and function of these cells upon its activation. After the implantation of the NDES into a 4T1 orthotopic mammary carcinoma model, both drugs were constantly released in a sustained manner by passive diffusion from the nanodevice into the TME. Local and systemic antitumor immune response was potentiated and tumor growth was inhibited by this nanotherapy, which exhibited higher antitumor efficacy than conventional immunotherapy [133].

## 4. Conclusions and Future Directions

**Figure 3:**
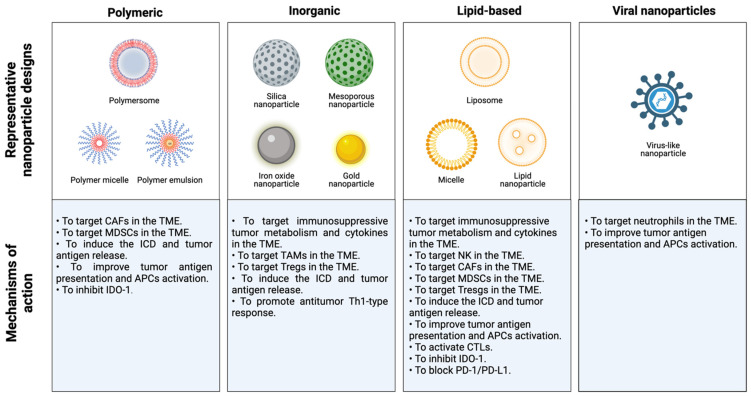
Representative classes of nanoparticle designs and mechanisms of action to overcome mechanisms of cancer immune escape. CAFs: carcinoma-associated fibroblasts; TME: tumor microenvironment; MDSCS: myeloid-derived suppressor cells; ICD: immunogenic cell death; APCs: antigen-presenting cells; IDO-1: indoleamine 2,3-dioxygenase 1; TAMs: tumor-associated macrophages; Tregs: regulatory T cells; NK: natural killer; CTLs: cytotoxic T lymphocytes; PD-1: programmed cell death protein 1; PD-L1: programmed cell death ligand 1.

Nanomedicine has gained great importance in cancer immunotherapy, as many emerging nanoplatforms with different designs have been recently developed to overcome various mechanisms of cancer immune escape and increase the efficacy of antitumor immune response (Figure 3).

In this regard, nanotherapy has certain advantages over conventional treatments. Nevertheless, this area still has a long way to go and it is necessary to address some challenges before its clinical translation to treat human breast cancer. To date, less than 20 nanoformulations (liposome-based, albumin-bound, iron-based, etc.) have been approved by the Food and Drug Administration (FDA) or the European Medicines Agency (EMA) for the treatment of solid tumors, including breast cancer [141,142]. This fact is in sharp contrast with the wide variety and number of investigations that report novel nanoformulations with antitumor efficacy and immunomodulatory functions. Here, we summarize and pinpoint some critical aspects that could be negatively influencing the lack of success in translating the research advances into the clinics.

Firstly, the formulation and engineering of nanosystems should allow large-scale, reproducible and profitable manufacturing [143,144]. Secondly, it has been reported that the physicochemical properties of NPs (shape, size, charge and surface coating) influence tissue biodistribution and cellular uptake. In this sense, the NPs’ shape is one of the surface properties that could affect the blood circulation time, cellular uptake and immune response of cancer nanotherapy. For instance, the spherical shape of NPs could preferentially generate a Th1-type immune response and increase antibody and proinflammatory cytokine production [145,146]. Regarding cellular uptake, rod and filamentous shapes might have better biodistribution than other shapes [147], while elongated NPs normally avoid phagocytosis and exhibit longer circulation times. Another important parameter that can dramatically affect in vivo distribution, clearance, cellular uptake and the induction of immune response is the NPs’ size. In general, it has been observed that NPs of 10–100 nm are able to extravasate from leaky tumor vasculature and accumulate more specifically in tumor tissues. On the contrary, NPs that are larger than 100 nm show poorer penetration into the tumor parenchyma, which restricts tumor accumulation. It should be noted that NPs with diameters smaller than 5–6 nm are rapidly removed by renal clearance, NPs above 200 nm in diameter are removed by liver and spleen clearance and larger NPs are mostly removed by the mononuclear phagocytic system [148]. Moreover, depending on the NPs’ size, different immune responses may be induced. In this context, 40 nm NPs have been observed to promote Th1-type and CD8 + T cell responses, while 100 nm NPs induce the Th2-type response [149]. Regarding the connection between NPs’ size and their effects on the immune system, it has been reported that controlling the diameter of NPs can lead to the distribution of nanocarriers to tissues enriched with APCs, which is especially relevant in developing cancer vaccines. After the systemic injection in mouse models, small NPs with a diameter of 70 nm were observed to preferentially accumulate in the lymph nodes, which facilitates their interaction and internalization by APCs and improves the therapeutic efficacy of nanovaccines [19]. The surface of the NPs is the third design parameter that can be tuned to enhance circulation half-life and tumor specificity, which further modulates their toxicity and the immune response. Thus, neutral or negatively charged NPs show longer blood circulation times, while the positive charge of NPs facilitates their interaction with cell membranes, which further improves the drug internalization of tumor cells [148,150]. Surface-charge-switchable NPs have been reported to enable a prolonged circulation time and specific drug delivery to the tumor site [76]. To increase serum stability and circulation time, the surfaces of NPs can be coated with hydrophilic polymers or biomimetic surfaces. Furthermore, specific ligands can be introduced onto the surfaces of NPs to enhance specific delivery to tumor tissues and reduce systemic toxicity [148,150]. Because it seems to be clear that the physicochemical properties of NPs are key factors that determine the interaction of nanocarriers with the immune system and modulate drug delivery in solid tumors [149], it is critical to question the translational impact of novel materials, whose systemic toxicity, metabolism, body clearance or compatibility with conventional therapies are not completely understood. We suggest that scientists should more deeply investigate how to fine-tune the physicochemical factors of NPs and understand how different compositions, geometries and surface modifications of nanocarriers can enhance their intrinsic immunomodulatory properties, prolong their circulation times and strengthen their antitumor effects, with the aim of ensuring translation into the clinical setting.

Thirdly, variations between the TME features of different types of tumors should also be evaluated to increase NPs’ delivery and optimize the choice of treatment for each type of cancer. Solid tumors, such as breast tumors, are characterized by a dysfunctional tumor vasculature that reduces tumor blood flow and hinders the distribution of NPs from blood vessels to cancer cells. Indirectly, the poor tumor blood flow also promotes immunosuppression in this area and reduces the responsiveness of these patients to nanomedicines. Hence, the normalization of the TME in solid tumors could significantly enhance intratumor drug penetration. The combination of immunotherapy with direct or indirect antiangiogenic therapies (AATs) has been proposed to normalize the morphology and function of tumor vasculature and improve tumor perfusion [19]. Indeed, several preclinical studies have shown that therapy with AATs can increase the antitumor potency of ICB agents [151,152], cancer vaccines [153] and adoptive cell therapy [154]. A promising alternative that should be more deeply exploited to treat solid tumors and their metastases is the normalization of the TME by the administration of nanotherapy after radiotherapy. This alternative has a dual effect because radiotherapy was demonstrated to improve the distribution of NPs in mouse models, and in turn, NPs could also perpetuate the abscopal effect of radiotherapy against distant metastases [19]. In conclusion, in order to better treat solid tumors such as breast cancer and enhance the relevance of nanomedicine in the clinical setting, we suggest to clinicians a personalized study of the TME in each patient and prior normalization, which would likely be necessary to promote drug penetration and reverse immunosuppression, thereby potentiating the effectiveness of the immunotherapies and conventional treatments delivered by nanocarriers in patients with solid tumors.

It was previously demonstrated that the dosing schedule, the timing of administration and the combination of nanotherapies must be individually optimized for patients in order to efficiently translate nanomedicine to the clinic [155]. In this sense, another important aspect that should be pointed out is that the use of novel NPs could be associated with health risks and there is an urgent need to overcome them, since the current knowledge about NPs’ accumulation, toxicity and pharmacokinetic behavior in humans is insufficient. In fact, we noticed that most of reports about novel materials reviewed herein lack of appropriate experimental approaches to understand and predict potential health issues in humans. In this regard, many studies have shown that NPs can induce toxicity, such as metal NPs, which exhibit reproductive and developmental toxicity, genotoxicity, as well as anxiogenic and depression effects and cause DNA damage via inflammation. Lipid NPs mainly cause anaphylactic and hypersensitivity reactions, while the damage caused by polymeric NPs is mainly attributed to the stabilizers used in their preparation, although they can also present systemic toxicity by themselves. Other studies show that silica NPs produce neurotoxicity, pulmonary diseases and impact on the immune system and virus-derived NP have mainly been associated with immunogenicity and systemic toxicity [156,157]. There are several strategies aimed at reducing the toxicity, immunogenicity and off-target effects exerted by NPs, which include PEGylation or coating with natural materials such as albumin or cell membranes [150]. However, other clinically relevant alternatives such as evaluating the route of administration according to the type of NP should be fully understood and addressed to reduce off-target effects. For example, nanocarriers inhaled or subcutaneously, intramuscularly and intranodally administered are capable of targeting lymph nodes and DCs, while orally administered NPs mainly target M cells in the gastrointestinal tract [19,158]. Furthermore, the metabolism of NPs is another important potential inductor of toxicity that must be addressed. In general, it is known that NPs made of biodegradable materials, such as PLGA, are easily metabolized and their effect on human health is known; however, metal-based NPs, such as gold-NPs, remain in the liver of mice for more than 6 months after their intravenous injection [158]. It has been observed that gold-NPs could accumulate primarily in the Kupffer cells in the liver of mice. Although gold-NPs seem to be inert and do not cause negative long-term effects in vivo after their accumulation in liver, there are some discrepancies between different studies. It is thought that modifications of the surface characteristics of the gold-NPs could impair their recognition by the Kupffer cells or even facilitate their binding to blood cells, thus reducing their accumulation in the liver [159]. For instance, the core-shell gold nanocage@manganese dioxide (AuNC@MnO2, AM) NPs were intravenously injected into a breast cancer murine model, and Au and Mn concentration levels in major organs (liver, spleen, lung and kidney) and tumors increased dramatically in 3 h in the murine model, but quickly decreased in 24 h and reached low levels in 21 days. Regarding systemic toxicity, these NPs did not cause obvious liver or renal toxicity and major organs did not show any histopathological abnormalities. Thus, the authors concluded that the nanoplatform was biodegradable without long-term safety concern. However, the metabolism pathway of the NPs was not described in detail [57]. Some questions arise according to these premises; for instance, can gold-NPs be metabolized? Very few studies discuss the metabolism of their NPs in murine models and offer limited information about the mechanisms of degradation, which could be one of the main reasons why those NPs have not moved into clinics yet. In addition, does the accumulation of those NPs have a negative impact on human health at long-term? Are the periods of time used to evaluated NPs toxicity appropriate to study long-term effects in vivo? NPs also undergo certain physicochemical changes when administered in vivo that make their metabolization even more unreliable and, therefore, the long-term effects of NPs’ accumulation are harder to predict [158]. Overall, we suggest that NPs made of novel materials should be proven to be safe to human health after long-term exposure, and scientists should provide exhaustive analyses of potential toxicities after NPs’ accumulation, as well as related solutions.

Based on these shortcomings of NPs, we propose cell-membrane-coating or biomimetic nanomedicine as a promising alternative. Specifically, white blood cell (WBC) membrane-coated nanosystems have unique properties that make their use for drug delivery worthwhile. For instance, these nanoplatforms facilitate a sustained circulation of drugs, since they are less subject to antibody opsonization and serum protein adsorption, exhibit superior tumor targeting (as they can target the sites of inflammation found in many solid tumors, including breast tumors) and are able to directly interact with cancer cells via specific surface receptors and ligands, which could enhance the therapeutic efficacy and reduce the side effects of WBC-membrane-coated NPs. Considering all these advantages, biomimetic nanoplatforms should be studied in the future to be implemented in breast cancer therapy [160]. Despite their benefits, cell membrane-coated NPs also have some drawbacks, such as the reproducibility of the coating procedure, which requires the development of adjustable methodologies, the lack of controls to ensure that the coated nanoparticles are not contaminated with microorganisms, and the complex protocols of membrane collection and purification, which require large numbers of cells. In fact, cell number might be one of the main problems to solve when coating with membranes from patient-derived cells, other than erythrocytes, not only due to the low number of cells, but also because membranes from immortalized cells are being coated onto NPs, which could lead to problems of immunogenicity or other undesirable/unknown effects. Therefore, to reduce side effects, ensure maximal compatibility when infused into cancer patients, and remain focused on personalized medicine, we suggest that biomimetic NPs derived from autologous cells would be a promising strategy to treat solid tumors. Nevertheless, efforts must be made to optimize the coating process with membranes derived from these kinds of cells [161].

Finally, to increase the therapeutic efficacy of NP-mediated cancer immunotherapy, the employment of several nanomedicine-based approaches to simultaneously target different mechanisms of immune escape in cancer should also be considered. For instance, antitumor T-cell response is altered by several immunosuppressive cells and cytokines within the TME, as well as through the interaction between the immune checkpoint molecules expressed by T cells and cancer cells. Therefore, the combination of different nanotherapies to simultaneously target different factors of tumor immunosuppression will have synergistic effects and evoke stronger antitumor T-cell responses. As a result, the inhibition of tumor growth and the therapeutic efficacy of combined treatments might be greater.

In this context, the efficacy of novel NPs was analyzed in vivo using 4T1 tumor-bearing mice in all of the aforementioned studies. Hence, it should be questioned whether using a unique murine model is appropriate to represent all the different immunological dynamics and changes of the TME that drive tumor progression in humans. Studies that use different experimental murine models may better reflect the TME of human breast tumors and might offer information about the success rates, pharmacokinetics and toxicity of novel therapies with a higher translational value to humans. Additionally, murine models are not able to demonstrate the transformation of tumors mediated by the immunoediting process that occurs in the human body through long periods of time, even decades. Therefore, the long-term effects of nanotherapies on tumor immune escape may not be completely studied using these murine models. Furthermore, the immunoediting process is continuously modifying the TME, making changes in the infiltrated populations of immune cells and varying the proportion of HLA-I-expressing cancer cells within the tumor. As a result, tumor expression of HLA-I is altered during different phases of the disease and the above-mentioned nanotherapies might lose effectiveness over time due to the loss of tumor HLA-I expression during tumor progression.

In that sense, it should be pointed out that the alteration of tumor HLA-I expression is a major issue, that is quite common in patients with breast cancer, due to which the response rates of immunotherapy are not as high as it was expected. Notably, most of the aforementioned nanotherapeutic approaches are based on the enhancement of tumor antigen presentation to trigger the antitumor CTL response. However, these strategies will not be effective if the tumor HLA-I complex is altered, as tumor antigens will not be presented to T cells even if they are activated. Considering this, evaluating this mechanism of immune escape may be crucial to improve immunotherapy outcomes in breast cancer patients. Nevertheless, to our knowledge, not a single nanomedicine strategy has been still designed to effectively target or recover HLA-I expression in cancer cells. Furthermore, none of the previous approaches evaluated the impact of tumor HLA-I expression on treatment efficacy. Importantly, patients with reversible tumor HLA-I alterations can recover in response to immunotherapies, whereas those who have irreversible defects may not significantly benefit from these treatments. Therefore, the analysis of tumor HLA-I expression is a potential biomarker tool for patients to select the most effective treatment. Thus, studying tumor HLA-I expression should also be taken into account in the development of innovative nanomedicine approaches, in order to take the most advantage of their immunotherapeutic potential. In addition, HLA-I independent nanotherapies, such as strategies based on CAR-T cells or NK cells, should also be improved in future studies, as their therapeutic efficacy may not be affected by the loss of HLA-I expression during the evolution of cancer.

In conclusion, numerous nanomedical approaches have been demonstrated to effectively override many mechanisms of immune escape in breast cancer and enhance the efficacy of the existing therapies. Even though the resolution of some drawbacks of nanomedicine is still necessary, the overcoming of tumor immune escape using nanomedicine offers great preliminary promise. Therefore, nanomedicine provides the opportunity to dramatically improve cancer immunotherapy and opens the door to a novel and revolutionary concept of breast cancer treatment.

## Figures and Tables

**Table 1:** Current applications of nanomedicine to overcome immunosuppression in the TME in patients with breast cancer.

Mechanism of Immune Escape as Therapeutic Target	Strategy to Overcome Immunosuppression in the TME	Applications of Nanomedicine to Ameliorate Cancer Immune Escape
Intrinsic immunosuppression of tumor cells	Inhibition of immunosuppressive cytokines	Nanodelivery of IL-10 protein trap [55]
	Specific nanodelivery of drugs in the acidic tumor area	Manganese dioxide nanoshells that release Ce6 and Dox under tumor acidic pH [56] Core-shell gold nanocage NPs that release @manganese dioxide under tumor acidic pH [57]
	Neutralization of tumor acidity	Nanodelivery of siRNA by cationic lipid-assisted NPs to knockdown lactate dehydrogenase A in tumor cells [58]
Immunosuppressive NK cells within the TME	Promotion of antitumor activity of NK cells	Extracellular vesicles derived from human NK cells pre-exposed to IL-15 [59]
Immunosuppressive macrophages within the TME	Repolarization of M2-like TAMs to the M1 phenotype	Nanodelivery of CSF1R and MAPK inhibitors into TAMs [60] Iron oxide NPs (ferumoxytol) targeting TAMs [61]
	Enhancement of antitumor macrophage activity	Delivery of macrolides into TAMs by colloidal gold nanorods [62]
Immunosuppressive CAFs within the TME	Inactivation of CAFs	Puerarin nanoemulsion [63] Injectable hydrogel carrying losartan [64]
Immunosuppressive MDSCs within the TME	Depletion of MDSCs in the TME	Syringeable immunomodulatory multidomain nanogel containing clodronate, GEM and R837 [65]
		Implantable synthetic immune niche containing GEM and a cancer vaccine [66]
		Liposomal nano-formulation of HER2/neu-derived P5 peptide and PEGylated liposomal Dox [67]
		Dox-polyglycerol-nanodiamond conjugate [68]
Immunosuppressive Tregs within the TME	Depletion of Tregs	Ursolic acid liposomes [69]
		Iron-oxide NPs [70]
		Zoledronic acid containing-NPs [71]
		NPs carrying an immunostimulant-invariant natural killer T-cell agonist and a selective inhibitor of the PI3K p110δ isoform [72]
Impairment of DCs’ activity and antigen presentation	Enhancement of tumor recognition by the induction of ICD of cancer cells	Zn-pyrophosphate shell NPs containing pyrolipid PS [73]
		Tumor-targeted polypyrrole NP with camptothecin and a near-infrared dye [74]
		Polydopamine nanomedicine that delivers a fluorescent agent and the TLR7/8 agonist R848 [75]
		TME-activatable vesicles carrying OXA prodrug and a PEGylated PS [76]
		Acidity-responsive nanocarrier that releases siCD47 into tumor cells and CCL25 protein in the tumor stroma [77]
		Highly integrated mesoporous silica NPs carrying Dox [78]
		Cancer cell membrane-coated calcium carbonate NPs containing low-dose Dox and Ce6 [79]
		NPs with a superior photothermal conversion efficacy carrying a PS agent and R837 [80]
		Poly(lactic-co-glycolic) acid-NPs that release a photothermal agent together with R837 [81]
Impairment of DCs’ activity and antigen presentation	Enhancement of tumor recognition by the induction of ICD of cancer cells	Low-molecular-weight heparin-d-α-tocopheryl succinate micelles carrying Dox and R837 [82] Immune nanoconverters carrying R848 and Dox [83] Coated with prodrug hyaluronic acid-Dox nanocores carrying R848 [84] Tumor-specific enhanced oxidative stress polymer conjugate to release QM and generate CA [85] Gold NP-based coat that delivers CpG-ODN and zinc phthalocyanine PS [86] Light-responsive chitosan-coated hollow CuS NPs assembling CpG-ODN [87]
	Potentiation of DC maturation	Polymeric cooper chelator RPTDH, pH-sensitive NPs carrying R848 [88]
	Enhancement of tumor antigen presentation	Peptide-based nanovaccines [89,90,91,92,93,94,95,96,97,98,99,100,101,102,103,104,105] Gene-based nanovaccines [106,107,108,109]
Impairment of antitumor T-cell response	Potentiation of T-cell activation	Synthetic multivalent antibodies retargeted exosomes (SMART-Exos) expressing on the surface anti-human CD3 and anti-human HER2 antibodies [110]
		DC-derived exosomes [111]
	Impairment of T-cell inactivation in the TME	IDO-1 inhibition [112,113,114,115,116,117,118,119,120]
		PD-1/PD-L1 blockade [121,122,123,124,125,126]
	Promotion of the Th1 response	Selenium NPs as nanovaccines [127,128,129,130,131]
		Chitosan-coated green synthesized copper oxide NPs with tumor lysate antigen [132]
		Nanofluidic-based drug eluting seed carrying aOX40 and CD40 monoclonal antibodies [133]

Ce6: chlorine e6; Dox: doxorubicin; NPs: nanoparticles; siRNA: small interference RNA; NK: natural killer; TME: tumor microenvironment; CSFR1: colony-stimulating factor 1 receptor; MAPK: mitogen-activated protein kinase; TAMs: tumor-associated macrophages; CAFs: carcinoma-associated fibroblasts; MDSCS: myeloid-derived suppressor cells; GEM: gemcitabine. Tregs: regulatory T cells; PI3K: phosphoinositide 3-kinase; DCs: dendritic cells; ICD: immunogenic cell death; PS: photosensitizer; OXA: oxaliplatin. QM: quinone methide; CA: cinnamaldehyde; CpG-ODN: oligodeoxynucleotides containing cytosine-guanine motifs; IDO-1: indoleamine 2,3-dioxygenase 1; PD-1: programmed cell death protein 1; PD-L1: programmed cell death ligand 1; Th1: type 1 T helper cells; aOX40: agonist tumor necrosis factor receptor superfamily member 4 antibody.

## References

[1] Ghoncheh M., Pournamdar Z., Salehiniya H. (2016). Incidence and mortality and epidemiology of breast cancer in the world. Asian Pac. J. Cancer Prev..

[2] Siegel R.L., Miller K.D., Jemal A. (2019). Cancer statistics, 2019. CA Cancer J. Clin..

[3] Adams S., Gatti-Mays M.E., Kalinsky K., Korde L.A., Sharon E., Amiri-Kordestani L., Bear H., McArthur H.L., Frank E., Perlmutter J. (2019). Current Landscape of Immunotherapy in Breast Cancer: A Review. JAMA Oncol..

[4] Steven A., Seliger B. (2018). The Role of Immune Escape and Immune Cell Infiltration in Breast Cancer. Breast Care.

[5] Monnot G.C., Romero P. (2018). Rationale for immunological approaches to breast cancer therapy. Breast.

[6] Couzin-Frankel J. (2013). Cancer immunotherapy. Science.

[7] Yang Y. (2015). Cancer immunotherapy: Harnessing the immune system to battle cancer. J. Clin. Investig..

[8] Christofi T., Baritaki S., Falzone L., Libra M., Zaravinos A. (2019). Current perspectives in cancer immunotherapy. Cancers.

[9] Criscitiello C., Curigliano G. (2013). Immunotherapeutics for breast cancer. Curr. Opin. Oncol..

[10] Emens L.A. (2012). Breast cancer immunobiology driving immunotherapy: Vaccines and immune checkpoint blockade. Expert Rev. Anticancer Ther..

[11] Lee Ventola C. (2017). Cancer immunotherapy, part 3: Challenges and future trends. Pharm. Therp..

[12] Sugie T. (2018). Immunotherapy for metastatic breast cancer. Chin. Clin. Oncol..

[13] Vonderheide R.H., Domchek S.M., Clark A.S. (2017). Immunotherapy for breast cancer: What are we missing?. Clin. Cancer Res..

[14] Stanton S.E., Adams S., Disis M.L. (2016). Variation in the Incidence and Magnitude of Tumor-Infiltrating Lymphocytes in Breast Cancer Subtypes: A Systematic Review. JAMA Oncol..

[15] Aaltomaa S., Lipponen P., Eskelinen M., Kosma V.M., Marin S., Alhava E., Syrjänen K. (1992). Lymphocyte infiltrates as a prognostic variable in female breast cancer. Eur. J. Cancer.

[16] Gu-Trantien C., Loi S., Garaud S., Equeter C., Libin M., De Wind A., Ravoet M., Le Buanec H., Sibille C., Manfouo-Foutsop G. (2013). CD4+ follicular helper T cell infiltration predicts breast cancer survival. J. Clin. Investig..

[17] Mahmoud S.M.A., Paish E.C., Powe D.G., Macmillan R.D., Grainge M.J., Lee A.H.S., Ellis I.O., Green A.R. (2011). Tumor-infiltrating CD8+ lymphocytes predict clinical outcome in breast cancer. J. Clin. Oncol..

[18] Mellman I., Coukos G., Dranoff G. (2011). Cancer immunotherapy comes of age. Nature.

[19] Martin J.D., Cabral H., Stylianopoulos T., Jain R.K. (2020). Improving cancer immunotherapy using nanomedicines: Progress, opportunities and challenges. Nat. Rev. Clin. Oncol..

[20] Retecki K., Seweryn M., Graczyk-Jarzynka A., Bajor M. (2021). The Immune Landscape of Breast Cancer: Strategies for Overcoming Immunotherapy Resistance. Cancers.

[21] Henriques B., Mendes F., Martins D. (2021). Immunotherapy in Breast Cancer: When, How, and What Challenges?. Biomedicines.

[22] García-Aranda M., Redondo M. (2019). Immunotherapy: A Challenge of Breast Cancer Treatment. Cancers.

[23] Cabrera T., Fernandez M.A., Sierra A., Garrido A., Herruzo A., Escobedo A., Fabra A., Garrido F. (1996). High frequency of altered HLA class I phenotypes in invasive breast carcinomas. Hum. Immunol..

[24] Garrido F., Cabrera T., Aptsiauri N. (2010). “Hard” and “soft” lesions underlying the HLA class I alterations in cancer cells: Implications for immunotherapy. Int. J. Cancer.

[25] Garrido M.A., Rodriguez T., Zinchenko S., Maleno I., Ruiz-Cabello F., Concha Á., Olea N., Garrido F., Aptsiauri N. (2018). HLA class I alterations in breast carcinoma are associated with a high frequency of the loss of heterozygosity at chromosomes 6 and 15. Immunogenetics.

[26] Seliger B., Maeurer M.J., Ferrone S. (2000). Antigen-processing machinery breakdown and tumor growth. Immunol. Today.

[27] Alsaab H.O., Sau S., Alzhrani R., Tatiparti K., Bhise K., Kashaw S.K., Iyer A.K. (2017). PD-1 and PD-L1 checkpoint signaling inhibition for cancer immunotherapy: Mechanism, combinations, and clinical outcome. Front. Pharmacol..

[28] Dong H., Strome S.E., Salomao D.R., Tamura H., Hirano F., Flies D.B., Roche P.C., Lu J., Zhu G., Tamada K. (2002). Tumor-associated B7-H1 promotes T-cell apoptosis: A potential mechanism of immune evasion. Nat. Med..

[29] Bertucci F., Finetti P., Colpaert C., Mamessier E., Parizel M., Dirix L., Viens P., Birnbaum D., Van Laere S. (2015). PDL1 expression in inflammatory breast cancer is frequent and predicts for the pathological response to chemotherapy. Oncotarget.

[30] Soliman H., Khalil F., Antonia S. (2014). PD-L1 Expression Is Increased in a Subset of Basal Type Breast Cancer Cells. PLoS ONE.

[31] Pinzon-Charry A., Ho C.S.K., Maxwell T., McGuckin M.A., Schmidt C., Furnival C., Pyke C.M., López J.A. (2007). Numerical and functional defects of blood dendritic cells in early- and late-stage breast cancer. Br. J. Cancer.

[32] Gorsch S.M., Memoli V.A., Stukel T.A., Gold L.I., Arrick B.A. (1992). Immunohistochemical Staining for Transforming Growth Factor β1 Associates with Disease Progression in Human Breast Cancer. Cancer Res..

[33] Groh V., Wu J., Yee C., Spies T. (2002). Tumour-derived soluble MIC ligands impair expression of NKG2D and T-cell activation. Nature.

[34] Mansfield A.S., Heikkila P., Von Smitten K., Vakkila J., Leidenius M. (2011). Metastasis to sentinel lymph nodes in breast cancer is associated with maturation arrest of dendritic cells and poor co-localization of dendritic cells and CD8+ T cells. Virchows Arch..

[35] Rubinstein N., Alvarez M., Zwirner N.W., Toscano M.A., Ilarregui J.M., Bravo A., Mordoh J., Fainboim L., Podhajcer O.L., Rabinovich G.A. (2004). Targeted inhibition of galectin-1 gene expression in tumor cells results in heightened T cell-mediated rejection: A potential mechanism of tumor-immune privilege. Cancer Cell.

[36] Dalotto-Moreno T., Croci D.O., Cerliani J.P., Martinez-Allo V.C., Dergan-Dylon S., Méndez-Huergo S.P., Stupirski J.C., Mazal D., Osinaga E., Toscano M.A. (2013). Targeting galectin-1 overcomes breast cancer-associated immunosuppression and prevents metastatic disease. Cancer Res..

[37] Uyttenhove C., Pilotte L., Théate I., Stroobant V., Colau D., Parmentier N., Boon T., Van den Eynde B.J. (2003). Evidence for a tumoral immune resistance mechanism based on tryptophan degradation by indoleamine 2,3-dioxygenase. Nat. Med..

[38] Mansfield A.S., Heikkila P.S., Vaara A.T., von Smitten K.A.J., Vakkila J.M., Leidenius M.H.K. (2009). Simultaneous Foxp3 and IDO expression is associated with sentinel lymph node metastases in breast cancer. BMC Cancer.

[39] Liyanage U.K., Moore T.T., Joo H.-G., Tanaka Y., Herrmann V., Doherty G., Drebin J.A., Strasberg S.M., Eberlein T.J., Goedegebuure P.S. (2002). Prevalence of Regulatory T Cells Is Increased in Peripheral Blood and Tumor Microenvironment of Patients with Pancreas or Breast Adenocarcinoma. J. Immunol..

[40] Wolf A.M., Wolf D., Steurer M., Gastl G., Gunsilius E., Grubeck-Loebenstein B. (2003). Increase of Regulatory T Cells in the Peripheral Blood of Cancer Patients. Clin. Cancer Res..

[41] Gunaydin G., Kesikli S.A., Guc D. (2015). Cancer associated fibroblasts have phenotypic and functional characteristics similar to the fibrocytes that represent a novel MDSC subset. Oncoimmunology.

[42] Kalluri R., Zeisberg M. (2006). Fibroblasts in cancer. Nat. Rev. Cancer.

[43] Treilleux I., Blay J.Y., Bendriss-Vermare N., Ray-Coquard I., Bachelot T., Guastolla J.P., Bremond A., Goddard S., Pin J.J., Bartfaelemy-Dubois C. (2004). Dendritic cell infiltration and prognosis of early stage breast cancer. Clin. Cancer Res..

[44] DeNardo D.G., Barreto J.B., Andreu P., Vasquez L., Tawfik D., Kolhatkar N., Coussens L.M. (2009). CD4+ T Cells Regulate Pulmonary Metastasis of Mammary Carcinomas by Enhancing Protumor Properties of Macrophages. Cancer Cell.

[45] Bronte V., Murray P.J. (2015). Understanding local macrophage phenotypes in disease: Modulating macrophage function to treat cancer. Nat. Med..

[46] Laoui D., Movahedi K., van Overmeire E., van den Bossche J., Schouppe E., Mommer C., Nikolaou A., Morias Y., de Baetselier P., van Ginderachter J.A. (2011). Tumor-associated macrophages in breast cancer: Distinct subsets, distinct functions. Int. J. Dev. Biol..

[47] Narita D., Seclaman E., Anghel A., Ilina R., Cireap N., Negru S., Sirbu I.O., Ursoniu S., Marian C. (2016). Altered levels of plasma chemokines in breast cancer and their association with clinical and pathological characteristics. Neoplasma.

[48] Tringler B., Zhuo S., Pilkington G., Torkko K.C., Singh M., Lucia M.S., Heinz D.E., Papkoff J., Shroyer K.R. (2005). B7-H4 is highly expressed in ductal and lobular breast cancer. Clin. Cancer Res..

[49] Khong H.T., Restifo N.P. (2002). Natural selection of tumor variants in the generation of “tumor escape” phenotypes. Nat. Immunol..

[50] Marincola F.M., Jaffee E.M., Hicklin D.J., Ferrone S. (1999). Escape of Human Solid Tumors from T–Cell Recognition: Molecular Mechanisms and Functional Significance. Adv. Immunol..

[51] (2008). Down-regulation of CD28, TCR-zeta (zeta) and up-regulation of FAS in peripheral cytotoxic T-cells of primary breast cancer patients. Anticancer. Res..

[52] Maeda H., Fang J., Inutsuka T., Kitamoto Y. (2003). Vascular permeability enhancement in solid tumor: Various factors, mechanisms involved and its implications. Int. Immunopharmacol..

[53] Maeda H. (2001). The enhanced permeability and retention (EPR) effect in tumor vasculature: The key role of tumor-selective macromolecular drug targeting. Adv. Enzyme Regul..

[54] Iyer A.K., Khaled G., Fang J., Maeda H. (2006). Exploiting the enhanced permeability and retention effect for tumor targeting. Drug Discov. Today.

[55] Shen L., Li J., Liu Q., Song W., Zhang X., Tiruthani K., Hu H., Das M., Goodwin T.J., Liu R. (2018). Local Blockade of Interleukin 10 and C-X-C Motif Chemokine Ligand 12 with Nano-Delivery Promotes Antitumor Response in Murine Cancers. ACS Nano.

[56] Yang G., Xu L., Chao Y., Xu J., Sun X., Wu Y., Peng R., Liu Z. (2017). Hollow MnO2 as a tumor-microenvironment-responsive biodegradable nano-platform for combination therapy favoring antitumor immune responses. Nat. Commun..

[57] Liang R., Liu L., He H., Chen Z., Han Z., Luo Z., Wu Z., Zheng M., Ma Y., Cai L. (2018). Oxygen-boosted immunogenic photodynamic therapy with gold nanocages@manganese dioxide to inhibit tumor growth and metastases. Biomaterials.

[58] Zhang Y.X., Zhao Y.Y., Shen J., Sun X., Liu Y., Liu H., Wang Y., Wang J. (2019). Nanoenabled Modulation of Acidic Tumor Microenvironment Reverses Anergy of Infiltrating T Cells and Potentiates Anti-PD-1 Therapy. Nano Lett..

[59] Zhu L., Kalimuthu S., Oh J.M., Gangadaran P., Baek S.H., Jeong S.Y., Lee S.W., Lee J., Ahn B.C. (2019). Enhancement of antitumor potency of extracellular vesicles derived from natural killer cells by IL-15 priming. Biomaterials.

[60] Ramesh A., Brouillard A., Kumar S., Nandi D., Kulkarni A. (2020). Dual inhibition of CSF1R and MAPK pathways using supramolecular nanoparticles enhances macrophage immunotherapy. Biomaterials.

[61] Zanganeh S., Hutter G., Spitler R., Lenkov O., Mahmoudi M., Shaw A., Pajarinen J.S., Nejadnik H., Goodman S., Moseley M. (2016). Iron oxide nanoparticles inhibit tumour growth by inducing pro-inflammatory macrophage polarization in tumour tissues. Nat. Nanotechnol..

[62] Dreaden E.C., Mwakwari S.C., Austin L.A., Kieffer M.J., Oyelere A.K., El-Sayed M.A. (2012). Small Molecule-Gold Nanorod Conjugates Selectively Target and Induce Macrophage Cytotoxicity towards Breast Cancer Cells. Small.

[63] Xu H., Hu M., Liu M., An S., Guan K., Wang M., Li L., Zhang J., Li J., Huang L. (2020). Nano-puerarin regulates tumor microenvironment and facilitates chemo- and immunotherapy in murine triple negative breast cancer model. Biomaterials.

[64] Hu C., Liu X., Ran W., Meng J., Zhai Y., Zhang P., Yin Q., Yu H., Zhang Z., Li Y. (2017). Regulating cancer associated fibroblasts with losartan-loaded injectable peptide hydrogel to potentiate chemotherapy in inhibiting growth and lung metastasis of triple negative breast cancer. Biomaterials.

[65] Song C., Phuengkham H., Kim Y.S., Dinh V.V., Lee I., Shin I.W., Shin H.S., Jin S.M., Um S.H., Lee H. (2019). Syringeable immunotherapeutic nanogel reshapes tumor microenvironment and prevents tumor metastasis and recurrence. Nat. Commun..

[66] Phuengkham H., Song C., Um S.H., Lim Y.T. (2018). Implantable Synthetic Immune Niche for Spatiotemporal Modulation of Tumor-Derived Immunosuppression and Systemic Antitumor Immunity: Postoperative Immunotherapy. Adv. Mater..

[67] Navashenaq J.G., Zamani P., Nikpoor A.R., Tavakkol-Afshari J., Jaafari M.R. (2020). Doxil chemotherapy plus liposomal P5 immunotherapy decreased myeloid-derived suppressor cells in murine model of breast cancer. Nanomed. Nanotechnol. Biol. Med..

[68] Yuan S.J., Xu Y.H., Wang C., An H.C., Xu H.Z., Li K., Komatsu N., Zhao L., Chen X. (2019). Doxorubicin-polyglycerol-nanodiamond conjugate is a cytostatic agent that evades chemoresistance and reverses cancer-induced immunosuppression in triple-negative breast cancer. J. Nanobiotechnol..

[69] Zhang N., Liu S., Shi S., Chen Y., Xu F., Wei X., Xu Y. (2020). Solubilization and delivery of Ursolic-acid for modulating tumor microenvironment and regulatory T cell activities in cancer immunotherapy. J. Control. Release.

[70] Chen H., Luan X., Paholak H.J., Burnett J.P., Stevers N.O., Sansanaphongpricha K., He M., Chang A.E., Li Q., Sun D. (2019). Depleting tumor-associated Tregs via nanoparticle-mediated hyperthermia to enhance anti-CTLA-4 immunotherapy. Nanomedicine.

[71] Kopecka J., Porto S., Lusa S., Gazzano E., Salzano G., Pinzòn-Daza M.L., Giordano A., Desiderio V., Ghigo D., De Rosa G. (2016). Zoledronic acid-encapsulating self-assembling nanoparticles and doxorubicin: A combinatorial approach to overcome simultaneously chemoresistance and immunoresistance in breast tumors. Oncotarget.

[72] Zhang F., Stephan S.B., Ene C.I., Smith T.T., Holland E.C., Stephan M.T. (2018). Nanoparticles that reshape the tumor milieu create a therapeutic window for effective T cell therapy in solid malignancies. Cancer Res..

[73] Duan X., Chan C., Guo N., Han W., Weichselbaum R.R., Lin W. (2016). Photodynamic Therapy Mediated by Nontoxic Core-Shell Nanoparticles Synergizes with Immune Checkpoint Blockade To Elicit Antitumor Immunity and Antimetastatic Effect on Breast Cancer. J. Am. Chem. Soc..

[74] Sun W., Du Y., Liang X., Yu C., Fang J., Lu W., Guo X., Tian J., Jin Y., Zheng J. (2019). Synergistic triple-combination therapy with hyaluronic acid-shelled PPy/CPT nanoparticles results in tumor regression and prevents tumor recurrence and metastasis in 4T1 breast cancer. Biomaterials.

[75] Lu Q., Qi S., Li P., Yang L., Yang S., Wang Y., Cheng Y., Song Y., Wang S., Tan F. (2019). Photothermally activatable PDA immune nanomedicine combined with PD-L1 checkpoint blockade for antimetastatic cancer photoimmunotherapy. J. Mater. Chem. B.

[76] Zhou F., Feng B., Yu H., Wang D., Wang T., Ma Y., Wang S., Li Y. (2019). Tumor Microenvironment-Activatable Prodrug Vesicles for Nanoenabled Cancer Chemoimmunotherapy Combining Immunogenic Cell Death Induction and CD47 Blockade. Adv. Mater..

[77] Chen H., Cong X., Wu C., Wu X., Wang J., Mao K., Li J., Zhu G., Liu F., Meng X. (2020). Intratumoral delivery of CCL25 enhances immunotherapy against triple-negative breast cancer by recruiting CCR9+ T cells. Sci. Adv..

[78] Zheng D.W., Chen J.L., Zhu J.Y., Rong L., Li B., Lei Q., Fan J.X., Zou M.Z., Li C., Cheng S.X. (2016). Highly Integrated Nano-Platform for Breaking the Barrier between Chemotherapy and Immunotherapy. Nano Lett..

[79] Ni J., Song J., Wang B., Hua H., Zhu H., Guo X., Xiong S., Zhao Y. (2020). Dendritic cell vaccine for the effective immunotherapy of breast cancer. Biomed. Pharmacother..

[80] Zhang L., Jing D., Wang L., Sun Y., Li J.J., Hill B., Yang F., Li Y., Lam K.S. (2018). Unique Photochemo-Immuno-Nanoplatform against Orthotopic Xenograft Oral Cancer and Metastatic Syngeneic Breast Cancer. Nano Lett..

[81] Chen Q., Xu L., Liang C., Wang C., Peng R., Liu Z. (2016). Photothermal therapy with immune-adjuvant nanoparticles together with checkpoint blockade for effective cancer immunotherapy. Nat. Commun..

[82] Wei J., Long Y., Guo R., Liu X., Tang X., Rao J., Yin S., Zhang Z., Li M., He Q. (2019). Multifunctional polymeric micelle-based chemo-immunotherapy with immune checkpoint blockade for efficient treatment of orthotopic and metastatic breast cancer. Acta Pharm. Sin. B.

[83] Phuengkham H., Song C., Lim Y.T. (2019). A Designer Scaffold with Immune Nanoconverters for Reverting Immunosuppression and Enhancing Immune Checkpoint Blockade Therapy. Adv. Mater..

[84] Liu Y., Qiao L., Zhang S., Wan G., Chen B., Zhou P., Zhang N., Wang Y. (2018). Dual pH-responsive multifunctional nanoparticles for targeted treatment of breast cancer by combining immunotherapy and chemotherapy. Acta Biomater..

[85] Ma S., Song W., Xu Y., Si X., Lv S., Zhang Y., Tang Z., Chen X. (2020). Rationally Designed Polymer Conjugate for Tumor-Specific Amplification of Oxidative Stress and Boosting Antitumor Immunity. Nano Lett..

[86] Marrache S., Choi J.H., Tundup S., Zaver D., Harn D.A., Dhar S. (2013). Immune stimulating photoactive hybrid nanoparticles for metastatic breast cancer. Integr. Biol..

[87] Guo L., Yan D.D., Yang D., Li Y., Wang X., Zalewski O., Yan B., Lu W. (2014). Combinatorial photothermal and immuno cancer therapy using chitosan-coated hollow copper sulfide nanoparticles. ACS Nano.

[88] Zhou P., Qin J., Zhou C., Wan G., Liu Y., Zhang M., Yang X., Zhang N., Wang Y. (2019). Multifunctional nanoparticles based on a polymeric copper chelator for combination treatment of metastatic breast cancer. Biomaterials.

[89] Wiedermann U., Wiltschke C., Jasinska J., Kundi M., Zurbriggen R., Garner-Spitzer E., Bartsch R., Steger G., Pehamberger H., Scheiner O. (2010). A virosomal formulated Her-2/neu multi-peptide vaccine induces Her-2/neu-specific immune responses in patients with metastatic breast cancer: A phase i study. Breast Cancer Res. Treat..

[90] Patel J.M., Vartabedian V.F., Kim M.C., He S., Kang S.M., Selvaraj P. (2015). Influenza virus-like particles engineered by protein transfer with tumor-associated antigens induces protective antitumor immunity. Biotechnol. Bioeng..

[91] Arab A., Behravan J., Razazan A., Gholizadeh Z., Nikpoor A.R., Barati N., Mosaffa F., Badiee A., Jaafari M.R. (2018). A nano-liposome vaccine carrying E75, a HER-2/neu-derived peptide, exhibits significant antitumour activity in mice. J. Drug Target..

[92] Zamani P., Navashenaq J.G., Teymouri M., Karimi M., Mashreghi M., Jaafari M.R. (2020). Combination therapy with liposomal doxorubicin and liposomal vaccine containing E75, an HER-2/neu-derived peptide, reduces myeloid-derived suppressor cells and improved tumor therapy. Life Sci..

[93] Razazan A., Behravan J., Arab A., Barati N., Arabi L., Gholizadeh Z., Hatamipour M., Nikpoor A.R., Momtazi-Borojeni A.A., Mosaffa F. (2017). Conjugated nanoliposome with the HER2/ neu-derived peptide GP2 as an effective vaccine against breast cancer in mice xenograft model. PLoS ONE.

[94] Zupančič E., Curato C., Kim J.S., Yeini E., Porat Z., Viana A.S., Globerson-Levin A., Waks T., Eshhar Z., Moreira J.N. (2018). Nanoparticulate vaccine inhibits tumor growth via improved T cell recruitment into melanoma and huHER2 breast cancer. Nanomed. Nanotechnol. Biol. Med..

[95] Kokate R.A., Chaudhary P., Sun X., Thamake S.I., Maji S., Chib R., Vishwanatha J.K., Jones H.P. (2016). Rationalizing the use of functionalized poly-lactic-co-glycolic acid nanoparticles for dendritic cell-based targeted anticancer therapy. Nanomedicine.

[96] Campbell D.F., Saenz R., Bharati I.S., Seible D., Zhang L., Esener S., Messmer B., Larsson M., Messmer D. (2015). Enhanced anti-tumor immune responses and delay of tumor development in human epidermal growth factor receptor 2 mice immunized with an immunostimulatory peptide in poly(d,l-lactic-co-glycolic) acid nanoparticles. Breast Cancer Res..

[97] Liu X., Feng Z., Wang C., Su Q., Song H., Zhang C., Huang P., Liang X.J., Dong A., Kong D. (2020). Co-localized delivery of nanomedicine and nanovaccine augments the postoperative cancer immunotherapy by amplifying T-cell responses. Biomaterials.

[98] Palladini A., Thrane S., Janitzek C.M., Pihl J., Clemmensen S.B., de Jongh W.A., Clausen T.M., Nicoletti G., Landuzzi L., Penichet M.L. (2018). Virus-like particle display of HER2 induces potent anti-cancer responses. Oncoimmunology.

[99] Cai H., Shukla S., Wang C., Masarapu H., Steinmetz N.F. (2019). Heterologous Prime-Boost Enhances the Antitumor Immune Response Elicited by Plant-Virus-Based Cancer Vaccine. J. Am. Chem. Soc..

[100] Bolli E., O’Rourke J.P., Conti L., Lanzardo S., Rolih V., Christen J.M., Barutello G., Forni M., Pericle F., Cavallo F. (2018). A Virus-Like-Particle immunotherapy targeting Epitope-Specific anti-xCT expressed on cancer stem cell inhibits the progression of metastatic cancer in vivo. Oncoimmunology.

[101] Alipour Talesh G., Ebrahimi Z., Badiee A., Mansourian M., Attar H., Arabi L., Jalali S.A., Jaafari M.R. (2016). Poly (I: C)-DOTAP cationic nanoliposome containing multi-epitope HER2-derived peptide promotes vaccine-elicited anti-tumor immunity in a murine model. Immunol. Lett..

[102] Shariat S., Badiee A., Jalali S.A., Mansourian M., Yazdani M., Mortazavi S.A., Jaafari M.R. (2014). P5 HER2/neu-derived peptide conjugated to liposomes containing MPL adjuvant as an effective prophylactic vaccine formulation for breast cancer. Cancer Lett..

[103] Zamani P., Navashenaq J.G., Nikpoor A.R., Hatamipour M., Oskuee R.K., Badiee A., Jaafari M.R. (2019). MPL nano-liposomal vaccine containing P5 HER2/neu-derived peptide pulsed PADRE as an effective vaccine in a mice TUBO model of breast cancer. J. Control. Release.

[104] Zamani P., Teymouri M., Nikpoor A.R., Navashenaq J.G., Gholizadeh Z., Darban S.A., Jaafari M.R. (2020). Nanoliposomal vaccine containing long multi-epitope peptide E75-AE36 pulsed PADRE-induced effective immune response in mice TUBO model of breast cancer. Eur. J. Cancer.

[105] Barati N., Nikpoor A.R., Razazan A., Mosaffa F., Badiee A., Arab A., Gholizadeh Z., Behravan J., Jaafari M.R. (2017). Nanoliposomes carrying HER2/neu-derived peptide AE36 with CpG-ODN exhibit therapeutic and prophylactic activities in a mice TUBO model of breast cancer. Immunol. Lett..

[106] Yamano T., Kaneda Y., Hiramatsu S.H., Huang S., Tran A.N., Giuliano A.E., Hoon D.S.B. (2007). Immunity against breast cancer by TERT DNA vaccine primed with chemokine CCL21. Cancer Gene Ther..

[107] Liu Z., Lv D., Liu S., Gong J., Wang D., Xiong M., Chen X., Xiang R., Tan X. (2013). Alginic Acid-Coated Chitosan Nanoparticles Loaded with Legumain DNA Vaccine: Effect against Breast Cancer in Mice. PLoS ONE.

[108] Liu L., Wang Y., Miao L., Liu Q., Musetti S., Li J., Huang L. (2018). Combination Immunotherapy of MUC1 mRNA Nano-vaccine and CTLA-4 Blockade Effectively Inhibits Growth of Triple Negative Breast Cancer. Mol. Ther..

[109] Jadidi-Niaragh F., Atyabi F., Rastegari A., Kheshtchin N., Arab S., Hassannia H., Ajami M., Mirsanei Z., Habibi S., Masoumi F. (2017). CD73 specific siRNA loaded chitosan lactate nanoparticles potentiate the antitumor effect of a dendritic cell vaccine in 4T1 breast cancer bearing mice. J. Control. Release.

[110] Shi X., Cheng Q., Hou T., Han M., Smbatyan G., Lang J.E., Epstein A.L., Lenz H.J., Zhang Y. (2020). Genetically Engineered Cell-Derived Nanoparticles for Targeted Breast Cancer Immunotherapy. Mol. Ther..

[111] Romagnoli G.G., Zelante B.B., Toniolo P.A., Migliori I.K., Barbuto J.A.M. (2015). Dendritic cell-derived exosomes may be a tool for cancer immunotherapy by converting tumor cells into immunogenic targets. Front. Immunol..

[112] Gao A., Chen B., Gao J., Zhou F., Saeed M., Hou B., Li Y., Yu H. (2020). Sheddable Prodrug Vesicles Combating Adaptive Immune Resistance for Improved Photodynamic Immunotherapy of Cancer. Nano Lett..

[113] Peng J., Xiao Y., Li W., Yang Q., Tan L., Jia Y., Qu Y., Qian Z. (2018). Photosensitizer Micelles Together with IDO Inhibitor Enhance Cancer Photothermal Therapy and Immunotherapy. Adv. Sci..

[114] Liu R., An Y., Jia W., Wang Y., Wu Y., Zhen Y., Cao J., Gao H. (2020). Macrophage-mimic shape changeable nanomedicine retained in tumor for multimodal therapy of breast cancer. J. Control. Release.

[115] Feng B., Hou B., Xu Z., Saeed M., Yu H., Li Y. (2019). Self-Amplified Drug Delivery with Light-Inducible Nanocargoes to Enhance Cancer Immunotherapy. Adv. Mater..

[116] Feng B., Zhou F., Hou B., Wang D., Wang T., Fu Y., Ma Y., Yu H., Li Y. (2018). Binary Cooperative Prodrug Nanoparticles Improve Immunotherapy by Synergistically Modulating Immune Tumor Microenvironment. Adv. Mater..

[117] Lu J., Liu X., Liao Y.P., Wang X., Ahmed A., Jiang W., Ji Y., Meng H., Nel A.E. (2018). Breast Cancer Chemo-immunotherapy through Liposomal Delivery of an Immunogenic Cell Death Stimulus Plus Interference in the IDO-1 Pathway. ACS Nano.

[118] Wan Z., Sun J., Xu J., Moharil P., Chen J., Xu J., Zhu J., Li J., Huang Y., Xu P. (2019). Dual functional immunostimulatory polymeric prodrug carrier with pendent indoximod for enhanced cancer immunochemotherapy. Acta Biomater..

[119] Sun J.J., Chen Y.C., Huang Y.X., Zhao W.C., Liu Y.H., Venkataramanan R., Lu B.F., Li S. (2017). Programmable co-delivery of the immune checkpoint inhibitor NLG919 and chemotherapeutic doxorubicin via a redox-responsive immunostimulatory polymeric prodrug carrier. Acta Pharmacol. Sin..

[120] Chen Y., Xia R., Huang Y., Zhao W., Li J., Zhang X., Wang P., Venkataramanan R., Fan J., Xie W. (2016). An immunostimulatory dual-functional nanocarrier that improves cancer immunochemotherapy. Nat. Commun..

[121] Tian Y., Wang X., Zhao S., Liao X., Younis M.R., Wang S., Zhang C., Lu G. (2019). JQ1-Loaded Polydopamine Nanoplatform Inhibits c-MYC/Programmed Cell Death Ligand 1 to Enhance Photothermal Therapy for Triple-Negative Breast Cancer. ACS Appl. Mater. Interfaces.

[122] Zhang R., Zhu Z., Lv H., Li F., Sun S., Li J., Lee C. (2019). Immune Checkpoint Blockade Mediated by a Small-Molecule Nanoinhibitor Targeting the PD-1/PD-L1 Pathway Synergizes with Photodynamic Therapy to Elicit Antitumor Immunity and Antimetastatic Effects on Breast Cancer. Small.

[123] Yu W., He X., Yang Z., Yang X., Xiao W., Liu R., Xie R., Qin L., Gao H. (2019). Sequentially responsive biomimetic nanoparticles with optimal size in combination with checkpoint blockade for cascade synergetic treatment of breast cancer and lung metastasis. Biomaterials.

[124] Lang T., Liu Y., Zheng Z., Ran W., Zhai Y., Yin Q., Zhang P., Li Y. (2019). Cocktail Strategy Based on Spatio-Temporally Controlled Nano Device Improves Therapy of Breast Cancer. Adv. Mater..

[125] Mi Y., Smith C.C., Yang F., Qi Y., Roche K.C., Serody J.S., Vincent B.G., Wang A.Z. (2018). A Dual Immunotherapy Nanoparticle Improves T-Cell Activation and Cancer Immunotherapy. Adv. Mater..

[126] Li G., Gao Y., Gong C., Han Z., Qiang L., Tai Z., Tian J., Gao S. (2019). Dual-Blockade Immune Checkpoint for Breast Cancer Treatment Based on a Tumor-Penetrating Peptide Assembling Nanoparticle. ACS Appl. Mater. Interfaces.

[127] Faghfuri E., Yazdi M.H., Mahdavi M., Sepehrizadeh Z., Faramarzi M.A., Mavandadnejad F., Shahverdi A.R. (2015). Dose-Response Relationship Study of Selenium Nanoparticles as an Immunostimulatory Agent in Cancer-bearing Mice. Arch. Med. Res..

[128] Yazdi M.H., Mahdavi M., Varastehmoradi B., Faramarzi M.A., Shahverdi A.R. (2012). The immunostimulatory effect of biogenic selenium nanoparticles on the 4T1 breast cancer model: An in vivo study. Biol. Trace Elem. Res..

[129] Yazdi M.H., Mahdavi M., Kheradmand E., Shahverdi A.R. (2012). The preventive oral supplementation of a selenium nanoparticle-enriched probiotic increases the immune response and lifespan of 4T1 breast cancer bearing mice. Arzneim. Forsch. Drug Res..

[130] Yazdi M.H., Mahdavi M., Setayesh N., Esfandyar M., Shahverdi A.R. (2013). Selenium nanoparticle-enriched Lactobacillus brevis causes more efficient immune responses in vivo and reduces the liver metastasis in metastatic form of mouse breast cancer. DARU J. Pharm. Sci..

[131] Yazdi M.H., Mahdavi M., Faghfuri E., Faramarzi M.A., Sepehrizadeh Z., Hassan Z.M., Gholami M., Shahverdi A.R. (2015). Th1 immune response induction by biogenic selenium nanoparticles in mice with breast cancer: Preliminary vaccine model. Iran. J. Biotechnol..

[132] Dey A., Manna S., Kumar S., Chattopadhyay S., Saha B., Roy S. (2020). Immunostimulatory effect of chitosan conjugated green copper oxide nanoparticles in tumor immunotherapy. Cytokine.

[133] Chua C.Y.X., Jain P., Susnjar A., Rhudy J., Folci M., Ballerini A., Gilbert A., Singh S., Bruno G., Filgueira C.S. (2018). Nanofluidic drug-eluting seed for sustained intratumoral immunotherapy in triple negative breast cancer. J. Control. Release.

[134] Huber V., Camisaschi C., Berzi A., Ferro S., Lugini L., Triulzi T., Tuccitto A., Tagliabue E., Castelli C., Rivoltini L. (2017). Cancer acidity: An ultimate frontier of tumor immune escape and a novel target of immunomodulation. Semin. Cancer Biol..

[135] Petrova V., Annicchiarico-Petruzzelli M., Melino G., Amelio I. (2018). The hypoxic tumour microenvironment. Oncogenesis.

[136] Lizotte P.H., Wen A.M., Sheen M.R., Fields J., Rojanasopondist P., Steinmetz N.F., Fiering S. (2016). In situ vaccination with cowpea mosaic virus nanoparticles suppresses metastatic cancer. Nat. Nanotechnol..

[137] Song Y., Tang C., Yin C. (2018). Combination antitumor immunotherapy with VEGF and PIGF siRNA via systemic delivery of multi-functionalized nanoparticles to tumor-associated macrophages and breast cancer cells. Biomaterials.

[138] Miao L., Wang Y., Lin C.M., Xiong Y., Chen N., Zhang L., Kim W.Y., Huang L. (2015). Nanoparticle modulation of the tumor microenvironment enhances therapeutic efficacy of cisplatin. J. Control. Release.

[139] Alizadeh D., Trad M., Hanke N.T., Larmonier C.B., Janikashvili N., Bonnotte B., Katsanis E., Larmonier N. (2014). Doxorubicin eliminates myeloid-derived suppressor cells and enhances the efficacy of adoptive T-cell transfer in breast cancer. Cancer Res..

[140] Patton D.T., Garden O.A., Pearce W.P., Clough L.E., Monk C.R., Leung E., Rowan W.C., Sancho S., Walker L.S.K., Vanhaesebroeck B. (2006). Cutting Edge: The Phosphoinositide 3-Kinase p110δ Is Critical for the Function of CD4 + CD25 + Foxp3 + Regulatory T Cells. J. Immunol..

[141] Anselmo A.C., Mitragotri S. (2019). Nanoparticles in the clinic: An update. Bioeng. Transl. Med..

[142] Mitchell M.J., Billingsley M.M., Haley R.M., Wechsler M.E., Peppas N.A., Langer R. (2020). Engineering precision nanoparticles for drug delivery. Nat. Rev. Drug Discov..

[143] Shukla S., Steinmetz N.F. (2016). Emerging nanotechnologies for cancer immunotherapy. Exp. Biol. Med..

[144] Yoon H.Y., Selvan S.T., Yang Y., Kim M.J., Yi D.K., Kwon I.C., Kim K. (2018). Engineering nanoparticle strategies for effective cancer immunotherapy. Biomaterials.

[145] Niikura K., Matsunaga T., Suzuki T., Kobayashi S., Yamaguchi H., Orba Y., Kawaguchi A., Hasegawa H., Kajino K., Ninomiya T. (2013). Gold nanoparticles as a vaccine platform: Influence of size and shape on immunological responses in vitro and in vivo. ACS Nano.

[146] Kumar S., Anselmo A.C., Banerjee A., Zakrewsky M., Mitragotri S. (2015). Shape and size-dependent immune response to antigen-carrying nanoparticles. J. Control. Release.

[147] Hashemzadeh N., Dolatkhah M., Adibkia K., Aghanejad A., Barzegar-Jalali M., Omidi Y., Barar J. (2021). Recent advances in breast cancer immunotherapy: The promising impact of nanomedicines. Life Sci..

[148] Zein R., Sharrouf W., Selting K. (2020). Physical Properties of Nanoparticles That Result in Improved Cancer Targeting. J. Oncol..

[149] Dobrovolskaia M.A., Shurin M., Shvedova A.A. (2016). Current understanding of interactions between nanoparticles and the immune system. Toxicol. Appl. Pharmacol..

[150] Jo S.D., Nam G.H., Kwak G., Yang Y., Kwon I.C. (2017). Harnessing designed nanoparticles: Current strategies and future perspectives in cancer immunotherapy. Nano Today.

[151] Schmittnaegel M., Rigamonti N., Kadioglu E., Cassará A., Rmili C.W., Kiialainen A., Kienast Y., Mueller H.J., Ooi C.H., Laoui D. (2017). Dual angiopoietin-2 and VEGFA inhibition elicits antitumor immunity that is enhanced by PD-1 checkpoint blockade. Sci. Transl. Med..

[152] Shigeta K., Datta M., Hato T., Kitahara S., Chen I.X., Matsui A., Kikuchi H., Mamessier E., Aoki S., Ramjiawan R.R. (2020). Dual Programmed Death Receptor-1 and Vascular Endothelial Growth Factor Receptor-2 Blockade Promotes Vascular Normalization and Enhances Antitumor Immune Responses in Hepatocellular Carcinoma. Hepatology.

[153] Huang Y., Yuan J., Righi E., Kamoun W.S., Ancukiewicz M., Nezivar J., Santosuosso M., Martin J.D., Martin M.R., Vianello F. (2012). Vascular normalizing doses of antiangiogenic treatment reprogram the immunosuppressive tumor microenvironment and enhance immunotherapy. Proc. Natl. Acad. Sci. USA.

[154] Shrimali R.K., Yu Z., Theoret M.R., Chinnasamy D., Restifo N.P., Rosenberg S.A. (2010). Antiangiogenic agents can increase lymphocyte infiltration into tumor and enhance the effectiveness of adoptive immunotherapy of cancer. Cancer Res..

[155] Qiu H., Min Y., Rodgers Z., Zhang L., Wang A.Z. (2017). Nanomedicine approaches to improve cancer immunotherapy. Wiley Interdiscip. Rev. Nanomed. Nanobiotechnol..

[156] Sharma S., Parveen R., Chatterji B.P. (2021). Toxicology of Nanoparticles in Drug Delivery. Curr. Pathobiol. Rep..

[157] Ganju A., Khan S., Hafeez B.B., Behrman S.W., Yallapu M.M., Chauhan S.C., Jaggi M. (2017). miRNA nanotherapeutics for cancer. Drug Discov. Today.

[158] Tyner K., Lee S., Wolfgang M., Li M., Zou P. (2016). Physiologically Based Pharmacokinetic (PBPK) Modeling of Pharmaceutical Nanoparticles. AAPS J..

[159] Sadauskas E., Danscher G., Stoltenberg M., Vogel U., Larsen A., Wallin H. (2009). Protracted elimination of gold nanoparticles from mouse liver. Nanomed. Nanotechnol. Biol. Med..

[160] Fang R.H., Kroll A.V., Gao W., Zhang L. (2018). Cell Membrane Coating Nanotechnology. Adv. Mater..

[161] Oroojalian F., Beygi M., Baradaran B., Mokhtarzadeh A., Shahbazi M.A. (2021). Immune Cell Membrane-Coated Biomimetic Nanoparticles for Targeted Cancer Therapy. Small.

